# Effects of Mixed-Strain Solid-State Fermentation on Mycotoxin Levels and Feeding Value of a Mixture Containing Spent *Flammulina velutipes* Substrate for Yellow-Feathered Broilers

**DOI:** 10.3390/ani16213363

**Published:** 2026-10-25

**Authors:** Jiaru Pei, Haorui Zhou, Xuelei Zhang, Xiaoxiao Liang, Yong Zhang

**Affiliations:** College of Biological Engineering, Henan University of Technology, Zhengzhou 450001, China; 2024930706@stu.haut.edu.cn (J.P.);

**Keywords:** spent *Flammulina velutipes* substrate, mycotoxin control, solid-state fermentation, nutritional quality, yellow-feathered broilers

## Abstract

Spent *Flammulina velutipes* substrate (SFVS) is nutritious, but its high crude fiber content and contamination with zearalenone (ZEN) and deoxynivalenol (DON) may limit feed use. This study optimized mixed-strain solid-state fermentation (SSF) of an SFVS-containing mixture and evaluated feeding value using 420 one-day-old male yellow-feathered broilers. Optimal conditions were a 2:1:2:3 inoculum ratio of *Saccharomyces cerevisiae*, *Bacillus coagulans*, *Clostridium butyricum*, and *Bacillus subtilis*, with 5-day fermentation, 47% moisture, and 7% inoculum. In the mixture, ZEN and DON concentrations decreased from 8622.43 and 1612.74 to 1349.82 and 750.21 μg/kg (84.35% and 53.48% reductions). Fermentation increased crude protein and total measured amino acids, decreased crude fiber (*p* < 0.01), and increased in vitro digestibilities of dry matter, crude protein, and crude fiber (*p* < 0.05). Over 42 d, 3% and 5% inclusion of the fermented mixture increased body weight (BW) on days 21 and 42 and average daily gain (ADG), while reducing feed conversion ratio (FCR) versus unfermented-mixture treatments (*p* < 0.05). At 3% and 5% inclusion, serum antioxidant status and jejunal morphology improved, while breast meat cooking loss and overall texture were not adversely affected; cecal communities were generally closer to the control (CON). The 7% inclusion provided no further benefit.

## Simple Summary

Spent *Flammulina velutipes* substrate (SFVS) is a by-product of edible mushroom production that retains nutritional value, but its relatively high fiber content and potential mycotoxin contamination may limit its use as a feed resource. In this study, mixed-strain solid-state fermentation was applied to a mixture containing SFVS, and its feeding effects were further evaluated in yellow-feathered broilers. The results showed that fermentation reduced the measured concentrations of zearalenone and deoxynivalenol in the mixture and improved the nutritional quality and digestibility of the mixture. Compared with the unfermented mixture, appropriate dietary inclusion of the fermented mixture generally supported better growth and feed utilization in yellow-feathered broilers and helped maintain antioxidant defenses and intestinal health, whereas a higher inclusion level provided no additional overall benefit. Overall, mixed-strain solid-state fermentation improved the feeding value of the fermented substrate mixture and may provide a practical approach for the sustainable use of mushroom-production by-products in poultry diets.

## 1. Introduction

Growing feed demand and the shift toward sustainable livestock production have driven the development and use of agricultural and food-processing by-products as unconventional feed resources. Using these by-products in feed production can improve resource-use efficiency and, to some extent, alleviate pressure on feed supplies [1]. The expansion of industrial mushroom cultivation has increased the need for effective disposal and resource utilization of spent cultivation substrates. Although spent mushroom substrate has been used as fertilizer, animal feed, and bioenergy feedstock, its large-scale and high-value utilization remains limited [2].

*Flammulina velutipes* is one of the edible mushrooms cultivated industrially in China, and the spent substrate generated during its large-scale production consists mainly of residual cultivation substrate and fungal mycelia [3]. Because part of the cultivation substrate remains incompletely utilized, spent *F. velutipes* substrate (SFVS) retains residual nutrients, including protein and amino acids. Previous studies have evaluated the feeding effects of SFVS and related products in broilers and reported effects on growth performance, small intestinal morphology, and cecal microbiota composition [4,5]. However, its relatively high fiber content and the potential risk of contamination with deoxynivalenol (DON) and zearalenone (ZEN) may still limit nutrient utilization and compromise its safety as a feed ingredient [6].

Solid-state fermentation (SSF) harnesses the functional characteristics of microorganisms to bioconvert solid substrates, thereby improving the nutrient composition and availability of agricultural by-products and enhancing their feeding value [7]. Given the multiple feeding constraints associated with SFVS, combining microorganisms with different functional characteristics in SSF may represent a potential strategy for reducing mycotoxin-related risks and improving substrate nutritional quality [8]. Previous studies have shown that the extracellular enzymatic activity of *Bacillus subtilis*, the adsorption capacity of β-D-glucans in the cell wall of *Saccharomyces cerevisiae*, and the inhibitory effects of *Bacillus coagulans* on *Fusarium* growth and DON production may all contribute to mycotoxin control [9,10,11]. In addition, the butyrate-producing capacity of *Clostridium butyricum* may contribute to modulation of the fermentation environment [12]. The differences in the functional characteristics of these four strains provided a rationale for developing a mixed-strain fermentation system designed to address mycotoxin-level control, nutrient transformation, and modulation of the fermentation environment.

Mixed-strain SSF has been investigated as an approach to improving the nutritional quality of food-processing by-products, including okara and brewer’s spent grain [13]. However, systematic studies on SFVS that use the concurrent reduction in ZEN and DON concentrations as an optimization objective and integrate fermentation process optimization, nutrient availability assessment, and broiler feeding evaluation remain limited. Therefore, the present study established and optimized a mixed-strain SSF system using a mixture containing SFVS as the substrate to evaluate the effects of fermentation on the mixture’s mycotoxin levels, nutritional quality, and in vitro digestibility, and further investigated the feeding effects of the fermented mixture in yellow-feathered broilers.

## 2. Materials and Methods

### 2.1. Preparation of the Fermentation Substrate

A single batch of SFVS was supplied by Xinxiang Xinghe Biotechnology Co., Ltd. (Xinxiang, Henan, China). It was generated after cultivation of *F. velutipes* on a substrate composed primarily of corncobs, soybean hulls, and rice husks. The initial substrate mixture was formulated with 80% SFVS, 7% molasses, 6% soybean meal, 6% wheat bran, and 1% fish meal (*w*/*w*). Molasses, soybean meal, wheat bran, and fish meal were purchased from Huabei Zhengda (Henan) Animal Husbandry Co., Ltd. (Zhengzhou, Henan, China). Within 3 h of collection, the SFVS was oven-dried. It was then thoroughly mixed with the other ingredients, and the resulting mixture was ground and passed through a 60-mesh sieve. The resulting mixture was stored in sealed containers in a cool, dry place until use. The fermented and unfermented substrate mixtures used in subsequent experiments were prepared from the same formulation described above. Before fermentation, samples of the formulated substrate mixture were analyzed for physicochemical properties and ZEN and DON concentrations.

### 2.2. Optimization of Mixed-Strain Solid-State Fermentation Conditions

*Saccharomyces cerevisiae* JGSW0229, *Bacillus coagulans* Nzrj360, *Clostridium butyricum* CGMCC No. 8187, and *Bacillus subtilis* CICC 22366 were supplied by Shanghai Jiaguan Biotechnology Co., Ltd. (Shanghai, China).

A weighted score based on the percentage reductions in ZEN and DON concentrations was used as the response variable for SSF optimization. A similar weighted scoring approach has previously been used to evaluate the reduction of multiple mycotoxins during solid-state fermentation [14]. Given that the initial ZEN concentration in the substrate mixture was higher than that of DON, relative weights of 0.6 and 0.4 were assigned to the percentage reductions in ZEN and DON concentrations, respectively, to evaluate the combined reduction of both mycotoxins. These weights were used for the overall evaluation of fermentation performance and were not determined based on the relative toxicological potencies of the two mycotoxins. The weighted score was calculated as follows:

Weighted score = 0.6 × percentage reduction in ZEN concentration + 0.4 × percentage reduction in DON concentration.

The optimal inoculum ratio of the four strains was determined using an L9(3^4^) orthogonal array and independently validated under the same fermentation conditions, with the weighted score calculated from the percentage reductions in ZEN and DON concentrations. Each strain was activated separately and cultured to the logarithmic growth phase, yielding microbial suspensions containing approximately 1 × 10^7^ CFU/mL. For each treatment, 200 g of the substrate mixture was inoculated with the corresponding microbial suspensions at the ratios specified in the experimental design. The total inoculum level and moisture content were set at 3% (*v*/*w*) and 45%, respectively. After thorough mixing, the substrate mixture was fermented in a 1 L glass fermentation vessel equipped with a one-way gas-release valve at 37 °C for 5 d, without active aeration. After fermentation, the fermented substrate mixture was oven-dried to constant weight; unfermented substrate mixture was subjected to the same drying procedure. ZEN and DON concentrations were then determined in the dried samples, and the percentage reductions in their concentrations and the combined weighted score were calculated.

With the optimal mixed-strain inoculum ratio held constant, total inoculum level (1%, 3%, 5%, 7%, and 9%; *v*/*w*), fermentation time (3, 5, 7, 9, and 11 d), and moisture content (30%, 35%, 40%, 45%, and 50%) were individually varied to assess their effects on the combined reduction in ZEN and DON concentrations. The ranges selected from the single-factor trials were used to construct a 3-factor, 3-level Box–Behnken design in Design-Expert 13 (version 13; Stat-Ease Inc., Minneapolis, MN, USA), and a quadratic regression model was fitted using the weighted score as the response variable (Table S1). Each treatment included 3 independent fermentation replicates.

### 2.3. Determination of ZEN and DON Concentrations and Percentage Reductions

The concentrations of ZEN and DON in the substrate mixture before and after fermentation were determined according to the instructions provided with the corresponding enzyme-linked immunosorbent assay (ELISA) kits (catalog nos. BA0741 and BA0941, respectively; Shanghai Youlong Biotechnology Co., Ltd., Shanghai, China). The assay sensitivities for ZEN and DON were 5 and 100 μg/kg, respectively. A calibration curve was generated using the corresponding standards for each kit (*R*^2^ > 0.99), and each sample was assayed in triplicate wells. The results were expressed on a dry matter (DM) basis. The percentage reduction in mycotoxin concentration was calculated as follows:

(1) $$\mathrm{Percentage}\;\mathrm{reduction}\;\mathrm{in}\;\mathrm{mycotoxin}\;\mathrm{concentration}\;(\%)\;=\;\frac{\left(C\mathrm{pre}-\;C\mathrm{post}\right)}{C\mathrm{pre}}\times 100,$$

where *C*_pre_ and *C*_post_ represent the mycotoxin concentrations before and after fermentation, respectively, expressed on a DM basis.

### 2.4. Determination of Nutrient Composition and In Vitro Digestibility

The pH, moisture content, crude protein (CP), ether extract (EE), crude fiber (CF), gross energy (GE), and amino acid composition of the substrate mixture before and after fermentation were determined according to the applicable standards listed in Table S2. Except for pH and moisture, the values for CP, EE, CF, GE, and amino acids were expressed on a DM basis. Total measured amino acid content was calculated as the sum of the contents of the 17 amino acids.

In vitro digestion was conducted using a poultry-specific simulated digestion kit (Hunan Zhongben Intelligent Technology Development Co., Ltd., Changsha, Hunan, China) following a previously described method [14]. DM, CP, and CF contents were determined in the samples before digestion and in the corresponding post-digestion residues. CP and CF contents were expressed on a DM basis. The fermented and unfermented substrate mixtures were each analyzed using 3 independent replicates. The corresponding in vitro digestibilities were calculated as follows:

(2) $$D_{X}\;(\%)\;=\;\frac{\left(\mathrm{Mx},\mathrm{before}-\mathrm{Mx},\mathrm{residue}\right)}{\mathrm{Mx},\mathrm{before}}\times 100,$$

where *D_X_* is the in vitro digestibility of component *X*; *M_X_*_,before_ and *M_X_*_,residue_ represent the masses of component *X* in the sample before digestion and in the post-digestion residue, respectively; and *X* represents DM, CP, or CF.

### 2.5. Determination of Apparent Metabolizable Energy

The animal procedures used in the apparent metabolizable energy assay were approved by the Animal Care and Use Committee of Henan University of Technology (approval no. HAUTETHI-2025-10-11). The apparent metabolizable energy (AME) of the fermented and unfermented substrate mixtures was determined using the force-feeding method with reference to GB/T 26437-2010 [15]. Healthy 20-wk-old Hy-Line Brown roosters weighing 2.0 ± 0.1 kg were purchased from a local poultry market and used for this determination. Each treatment comprised 4 replicates, with 1 rooster per replicate. The roosters were acclimated in metabolism cages for 7 d, and the difference in mean body weight (BW) between the 2 treatment groups did not exceed 100 g.

Before the assay, all roosters were fasted for 48 h with free access to water. After fasting, each rooster was force-fed 50 g of the corresponding test sample, and all excreta were subsequently collected for 48 h. The collected excreta were dried at 65 °C to constant weight, equilibrated at room temperature for 24 h, and then used for DM and GE determinations. The dry matter content of each test sample was used to calculate the actual dry matter intake. AME was calculated from the difference between GE intake and GE excreted and was expressed on a dry matter basis. Endogenous energy losses were not corrected for in the AME calculation.

### 2.6. Animal Experiment

All animal care and experimental procedures involved in the broiler feeding trial were approved by the Animal Care and Use Committee of Henan University of Technology (approval no. HAUTETHI-2025-1120). A total of 420 healthy 1-day-old male yellow-feathered broilers with an initial BW of 35 to 40 g were obtained from Xinzheng Lianhe Poultry Co., Ltd. (Xinzheng, Henan, China). The 42-day feeding period was divided into a starter phase (days 1–21) and a grower phase (days 22–42). After stratification by initial BW, the birds were randomly allocated to 7 dietary treatments. Each treatment included 6 replicate cages with 10 birds per cage. The treatments comprised a basal diet control (CON); diets containing 3%, 5%, or 7% fermented substrate mixture (3F, 5F, and 7F, respectively); and diets containing 3%, 5%, or 7% unfermented substrate mixture (3WF, 5WF, and 7WF, respectively).

The diets for both phases were formulated in accordance with GB/T 5916-2020 [16]. The ingredient composition and nutrient levels of each treatment are provided in Table 1. Birds were housed in three-tier cages, with the six replicate cages of each treatment evenly distributed across the three tiers (two replicate cages per tier). The poultry house was preheated to 35 °C 24 h before chick placement. Thereafter, the temperature was lowered by approximately 3 °C each week until it reached approximately 21 °C, and relative humidity was maintained at 55% to 65%. Throughout the trial, feed and water were provided ad libitum. Chicks were provided with approximately 23 h of light per day during the first 3 d after placement, after which the photoperiod was gradually shortened with age, reaching approximately 12 h of light and 12 h of darkness (12L:12D) by the end of week 6. The poultry house was cleaned regularly and kept adequately ventilated.

At the end of the trial, feed was withdrawn from all birds for 12 h. From each replicate cage, one bird with a BW closest to the cage mean was selected, giving 42 birds in total. The selected birds were anesthetized with sodium pentobarbital before blood collection from the wing vein and were subsequently euthanized by exsanguination after severing the cervical blood vessels in accordance with the approved animal ethics protocol. Major organs, relevant tissues, breast muscle, and cecal contents were then collected. Portions of liver and jejunal tissues were fixed in 4% paraformaldehyde, whereas separate liver, jejunal, and pancreatic tissue samples and cecal contents were snap-frozen in liquid nitrogen and stored at −80 °C until subsequent analysis. Breast muscle samples were stored at 4 °C, and meat quality measurements were completed within 48 h after collection. Serum was separated from the collected blood and stored at −80 °C until analysis.

### 2.7. Determination of Growth Performance and Relative Organ Weights

On days 1, 21, and 42, the total body weight of the broilers in each replicate cage was measured, and mean BW was calculated based on the actual number of birds present. Throughout the experiment, the amount of feed offered to each replicate cage was recorded, and feed refusals were collected and weighed weekly. Average daily feed intake (ADFI), average daily gain (ADG), and feed conversion ratio (FCR) were calculated for each replicate cage based on changes in body weight, the actual number of birds present, feed offered, and feed refusals. At the end of the trial, the heart, liver, spleen, kidneys, and bursa of Fabricius of the selected broilers were weighed. Relative organ weight was calculated as the ratio of organ weight to preslaughter BW and expressed as g/kg BW. No birds died or were excluded during the 42 d feeding trial, and the number of birds in each replicate cage remained constant at 10.

### 2.8. Meat Quality Analysis

After slaughter, the left pectoralis major muscle was collected and divided into separate portions for the determination of different meat quality parameters. Breast muscle pH, color, and cooking loss were determined according to NY/T 2793-2015 [17]. At 45 min, 24 h, and 48 h postmortem, muscle pH and color were measured using a portable pH meter (Testo 205; Testo AG, Lenzkirch, Germany) and a handheld colorimeter (LS173; Shenzhen Linshang Technology Co., Ltd., Shenzhen, Guangdong, China), respectively. Color was expressed as L* (lightness), a* (redness), and b* (yellowness) values. For cooking loss determination, the corresponding samples were heated in a water bath at 72 °C until the core temperature reached 70 °C. After cooling, the samples were weighed, and cooking loss was calculated from the change in sample weight before and after heating.

Shear force was determined using a texture analyzer (TA.XTC; Shanghai Bosin Industrial Development Co., Ltd., Shanghai, China) equipped with a TA/MORSE shear blade, following NY/T 1180-2006 [18]. The samples used for shear force and texture profile analysis (TPA) were heated in a water bath at 80 °C until the core temperature reached 70 °C and were then cooled until the core temperature reached 4 °C. For shear force measurement, cylindrical specimens with a diameter of 1.27 cm were prepared parallel to the muscle fiber direction using the dedicated coring device supplied with the texture analyzer. TPA was conducted using the same analyzer equipped with a TA36 probe, and the measured parameters included hardness, springiness, cohesiveness, gumminess, chewiness, and resilience. Each variable was measured in triplicate for each sample, and the mean was used as the sample value.

### 2.9. Determination of Serum Antioxidant Indices and Hepatic Enzyme Activities

Serum malondialdehyde (MDA) concentration and the activities of total superoxide dismutase (T-SOD), glutathione peroxidase (GSH-Px), and catalase (CAT) were determined using catalog nos. A003-1-2, A001-1-1, A005-1-2, and A007-1-1, respectively, from Nanjing Jiancheng Bioengineering Institute (Nanjing, Jiangsu, China). Hepatic alanine aminotransferase (ALT), aspartate aminotransferase (AST), and alkaline phosphatase (ALP) activities were measured using catalog nos. C009-1-1, C010-1-1, and A059-1-1, respectively, from the same manufacturer. Liver tissue was homogenized with physiological saline at a 1:9 (*w*/*v*) ratio in an ice-water bath. After centrifugation, the supernatant was collected for the determination of ALT, AST, and ALP activities. Total protein concentration in the liver homogenate supernatant was determined using the bicinchoninic acid (BCA) assay (catalog no. A045-3-2) and was used to normalize hepatic enzyme activities. MDA, T-SOD, GSH-Px, and ALP were determined by colorimetric assays using a microplate reader at the wavelengths specified by the respective kits, whereas CAT, ALT, and AST were determined by spectrophotometric assays using a spectrophotometer at the corresponding wavelengths. All assays were performed according to the kit instructions.

### 2.10. Histological Analysis

Liver and jejunal tissues were fixed in 4% paraformaldehyde and stained with hematoxylin and eosin (H&E) according to a previously described method [19]. Liver sections were used for histopathological examination, whereas jejunal sections were used for morphometric measurements. For histopathological examination, representative microscopic fields were selected from areas with intact tissue architecture and no obvious sectioning artifacts to assess the overall histological features of the liver. During jejunal morphometric analysis, villus–crypt units with intact villi and crypts, clear orientation, and no obvious sectioning artifacts were selected to minimize the effects of oblique sectioning, tissue folding, or tearing on the measurements. Five villus–crypt units meeting these criteria were selected from each jejunal sample. Villus height (VH) and crypt depth (CD) were measured using ImageJ software (version 2.16.0; National Institutes of Health, Bethesda, MD, USA), and the VH:CD ratio was calculated. Histopathological examination of liver sections and morphometric measurements of jejunal sections were performed with the observer blinded to treatment allocation.

### 2.11. Reverse Transcription and Quantitative PCR Analysis

Total RNA was extracted from jejunal and pancreatic tissues stored at −80 °C using FreeZol Reagent (catalog no. R711-01). RNA concentration and purity were measured with a NanoDrop 2000 microvolume spectrophotometer (Thermo Fisher Scientific, Waltham, MA, USA). Total RNA was reverse-transcribed into cDNA using HiScript III All-in-one RT SuperMix Perfect for qPCR (catalog no. R333-01). The quantitative PCR (qPCR) mixtures were prepared with Taq Pro Universal SYBR qPCR Master Mix (catalog no. Q712-02), and amplification was performed on an FQD-96C real-time PCR system (Hangzhou Bioer Technology Co., Ltd., Hangzhou, Zhejiang, China). All 3 reagents were obtained from Vazyme Biotech Co., Ltd. (Nanjing, Jiangsu, China). The amplification program consisted of 30 s at 95 °C, followed by 40 cycles of 10 s at 95 °C and 20 s at 60 °C. Melting-curve analysis was conducted after amplification. Each sample was analyzed in 3 technical replicates. *GAPDH* and *ACTB* were used as the reference genes for jejunal and pancreatic tissues, respectively. Relative mRNA expression of the target genes was calculated using the 2^−ΔΔ*C*t^ method. Primer sequences are listed in Table S3.

### 2.12. Analysis of Cecal Microbiota Structure and Diversity

Cecal contents were stored at −80 °C and processed by Shanghai Majorbio Bio-Pharm Technology Co., Ltd. (Shanghai, China) for microbial genomic DNA extraction, 16S rRNA gene amplicon sequencing, and downstream bioinformatics analysis. The V3–V4 region of the bacterial 16S rRNA gene was amplified with the 338F/806R primer pair, and the resulting libraries were sequenced on an Illumina MiSeq platform (Illumina, Inc., San Diego, CA, USA) using a paired-end 2 × 300 bp format. Sequencing reads were quality-filtered using fastp (10 bp sliding window and Q20 quality threshold; reads shorter than 50 bp or containing more than five N bases were removed), and paired-end reads were subsequently merged using FLASH with a minimum overlap length of 10 bp and a maximum mismatch ratio of 0.2. Following quality control and chimera removal, the sequences were grouped into operational taxonomic units (OTUs) at 97% sequence identity with UPARSE in USEARCH v11.0. Representative sequences were taxonomically assigned with the Ribosomal Database Project Classifier against SILVA release 138.2 using a confidence cutoff of 0.7.

Alpha-diversity indices, including the abundance-based coverage estimator (ACE), Chao1, Shannon, and Simpson indices, were calculated based on the OTU abundance table. Beta diversity was analyzed based on Bray–Curtis dissimilarity, and differences in microbial community structure among treatment groups were visualized using principal coordinate analysis (PCoA) and non-metric multidimensional scaling (NMDS). Differences in cecal microbial community structure among treatment groups were assessed using permutational multivariate analysis of variance (PERMANOVA) with 999 permutations. LEfSe (linear discriminant analysis effect size) analysis was used to identify differentially enriched taxa among treatment groups. Differential features were screened using the Kruskal–Wallis rank-sum test, and their effect sizes were evaluated using linear discriminant analysis (LDA). Significance results for LEfSe were adjusted for the false discovery rate (FDR), with an LDA score ≥ 2 as the criterion for selecting differential features. Associations between differentially abundant genera and growth performance, serum biochemical indices, meat quality traits, and intestinal gene expression were evaluated using Spearman’s rank correlation analysis. *p* values for all 84 genus–phenotype correlations were adjusted using the Benjamini–Hochberg procedure for FDR control, with an adjusted q value < 0.05 considered statistically significant.

### 2.13. Data Processing and Statistical Analysis

Normality and homogeneity of variance were assessed before parametric analyses. Except for the response surface experiment, data from fermentation-process optimization and the associated in vitro measurements were statistically analyzed using IBM SPSS Statistics 24.0 (IBM Corp., Armonk, NY, USA), with each independent replicate sample considered the experimental unit. Data from the single-factor experiments were analyzed by one-way analysis of variance, followed by Tukey’s multiple-comparison test. Data involving two-group comparisons were analyzed using *t*-tests. For the L9(3^4^) orthogonal experiment, range analysis was performed using the weighted score as the response variable. The *K*_1_–*K*_3_, *k*_1_–*k*_3_, and range (*R*) values for the 3 levels of each factor were calculated to evaluate the relative effects of the four strain-ratio factors on the weighted score and to determine the optimal level combination. For the response surface experiment, a quadratic regression model was established using Design-Expert 13, and analysis of variance was used to evaluate model significance and goodness of fit.

Animal experiment data were analyzed using SAS 9.2 (SAS Institute Inc., Cary, NC, USA). The replicate cage was considered the experimental unit for growth performance, whereas one representative broiler selected from each replicate cage served as the experimental unit for all other animal measurements, with 6 replicates per treatment. BW, breast muscle pH, and color measurements were analyzed separately at each measurement time point. Excluding the CON, the six dietary treatments containing the fermented or unfermented substrate mixture were analyzed using a 2 × 3 factorial model, with fermentation status (fermented vs. unfermented), dietary inclusion level (3%, 5%, and 7%), and their interaction included as fixed effects. Residual normality and homogeneity of variance were assessed using the Shapiro–Wilk and Levene tests, respectively. Appropriate data transformations were first applied when model assumptions were not adequately met. HC3-robust inference was used for heteroscedastic outcomes, whereas Freedman–Lane residual-permutation tests were applied when residual non-normality persisted despite acceptable variance homogeneity. Relative qPCR expression data were analyzed after log_2_ transformation. When the interaction was significant (*p* < 0.05), Tukey-adjusted multiple comparisons were performed among the least-squares means of the 6 treatment combinations for outcomes meeting conventional parametric assumptions, whereas Holm-adjusted comparisons were used for outcomes analyzed using robust or permutation procedures. When the interaction was not significant, the main effects of fermentation status and inclusion level were interpreted based on the corresponding tests in the factorial model. CON was not included in the 2 × 3 factorial model, and differences between each dietary treatment containing the fermented or unfermented substrate mixture and CON were evaluated separately using a one-way model including all seven groups. Dunnett’s test was used for outcomes meeting conventional parametric assumptions, with the pooled within-group residual mean square from this model as the error term. For heteroscedastic outcomes, Holm-adjusted HC3-robust contrasts were used, whereas Holm-adjusted residual-permutation contrasts were applied when residual non-normality persisted despite acceptable variance homogeneity. For the animal experiment tables, the standard error of the mean (SEM) represents the pooled standard error of a treatment-combination mean calculated from the residual mean square of the untransformed 2 × 3 factorial model.

Overall differences in cecal microbial community structure were assessed using permutational multivariate analysis of variance (PERMANOVA), and associations between differentially abundant bacterial genera and host phenotypic variables were evaluated using Spearman’s rank correlation analysis. Unless otherwise stated, data are presented as means ± SEM, and statistical significance was defined as *p* < 0.05.

## 3. Results

### 3.1. Optimization of Mixed-Strain Solid-State Fermentation Conditions and Changes in Mycotoxin Concentrations

Range analysis of the orthogonal experiment showed that the effects of the four strain-ratio factors on the weighted score followed the order B > A > C > D. The optimal level combination was A_2_B_1_C_2_D_3_, corresponding to an inoculum ratio of 2:1:2:3 for *Saccharomyces cerevisiae*, *Bacillus coagulans*, *Clostridium butyricum*, and *Bacillus subtilis*, respectively, and this combination yielded the highest weighted score among the nine tested combinations (Run 4; Table 2). An independent validation experiment conducted under the same fermentation conditions yielded a weighted score of 58.93 ± 0.18 (*n* = 3), which was close to that obtained in the orthogonal experiment. Therefore, this ratio was selected as the optimal mixed-strain inoculum ratio for subsequent optimization of the fermentation process.

**Figure 1:**
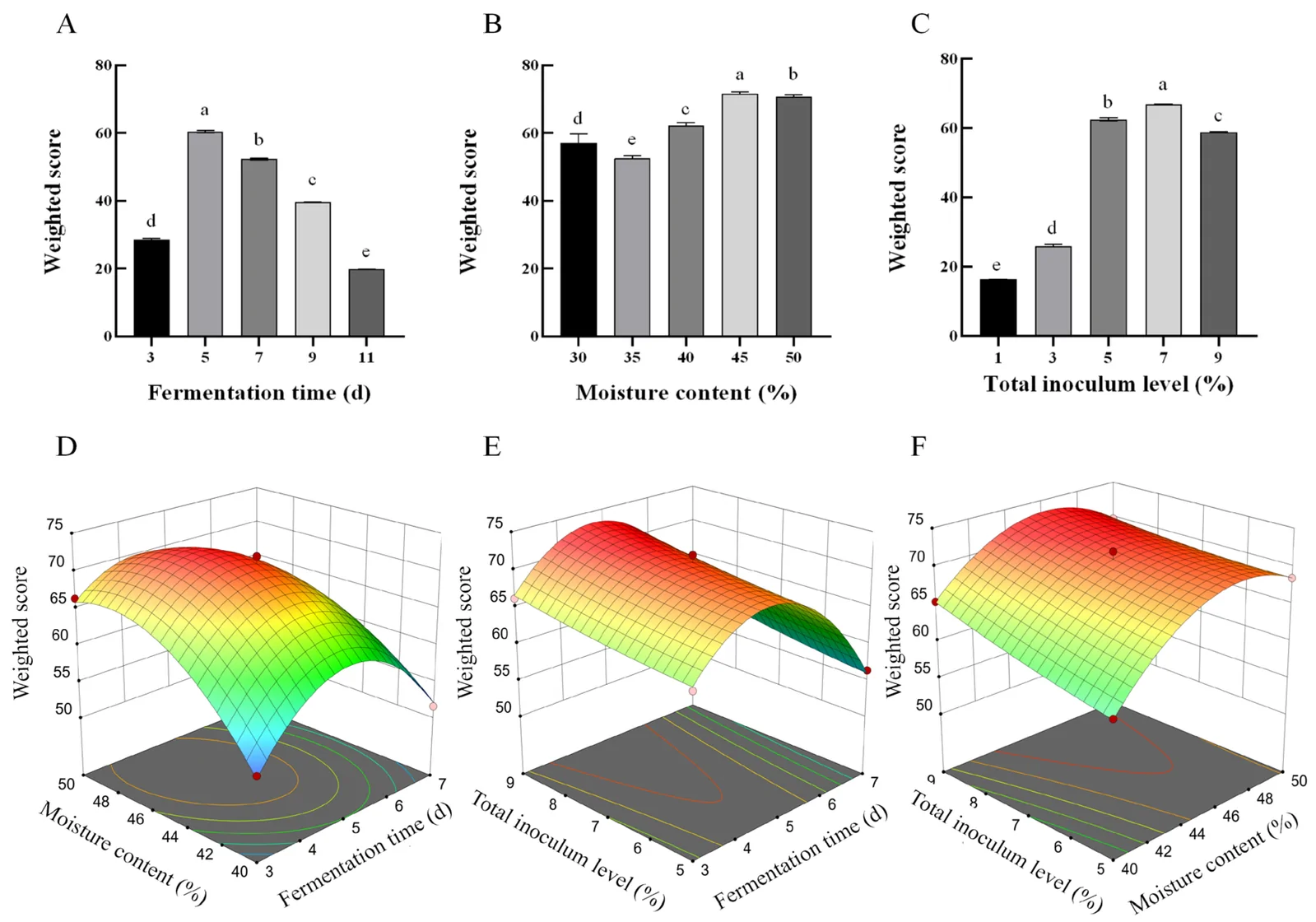
Optimization of mixed-strain solid-state fermentation conditions. Effects of fermentation time (**A**), moisture content (**B**), and total inoculum level (**C**) on the weighted score; response surface plots showing the interactions between moisture content and fermentation time (**D**), total inoculum level and fermentation time (**E**), and total inoculum level and moisture content (**F**). The weighted score was calculated as follows: weighted score = 0.6 × percentage reduction in ZEN concentration + 0.4 × percentage reduction in DON concentration. Data in (**A**–**C**) are presented as means ± SEM (*n* = 3 independent fermentation replicates). In (**A**–**C**), different lowercase letters indicate significant differences among levels of the same factor (*p* < 0.05). In (**D**–**F**), the color gradient represents the predicted weighted score, the points indicate experimental data points, and the contour lines on the base plane represent equal predicted weighted-score levels.

In the single-factor experiments, the weighted score varied with total inoculum level, fermentation time, and moisture content (Figure 1A–C). Relatively high weighted scores were observed at an inoculum level of 7%, a fermentation time of 5 d, and a moisture content of 45%. The fitted response surface model showed excellent fit and predictive performance (*R*^2^ = 0.9939, adjusted *R*^2^ = 0.9861, and predicted *R*^2^ = 0.9610; Table S4). Interactions were detected between fermentation time and moisture content and between total inoculum level and moisture content (*p* < 0.05), but not between fermentation time and total inoculum level (*p* > 0.05; Figure 1D–F). Numerical optimization yielded an optimal solution with a fermentation time of 4.65 d, a moisture content of 46.87%, and a total inoculum level of 7.18%, corresponding to a predicted weighted score of 72.12. Considering practical feasibility, the fermentation conditions were adjusted to a fermentation time of 5 d, a moisture content of 47%, and an inoculum level of 7%. The validation experiment yielded a weighted score of 72.00, which agreed closely with the predicted value of 72.12 and supported the predictive reliability of the model.

**Figure 2:**
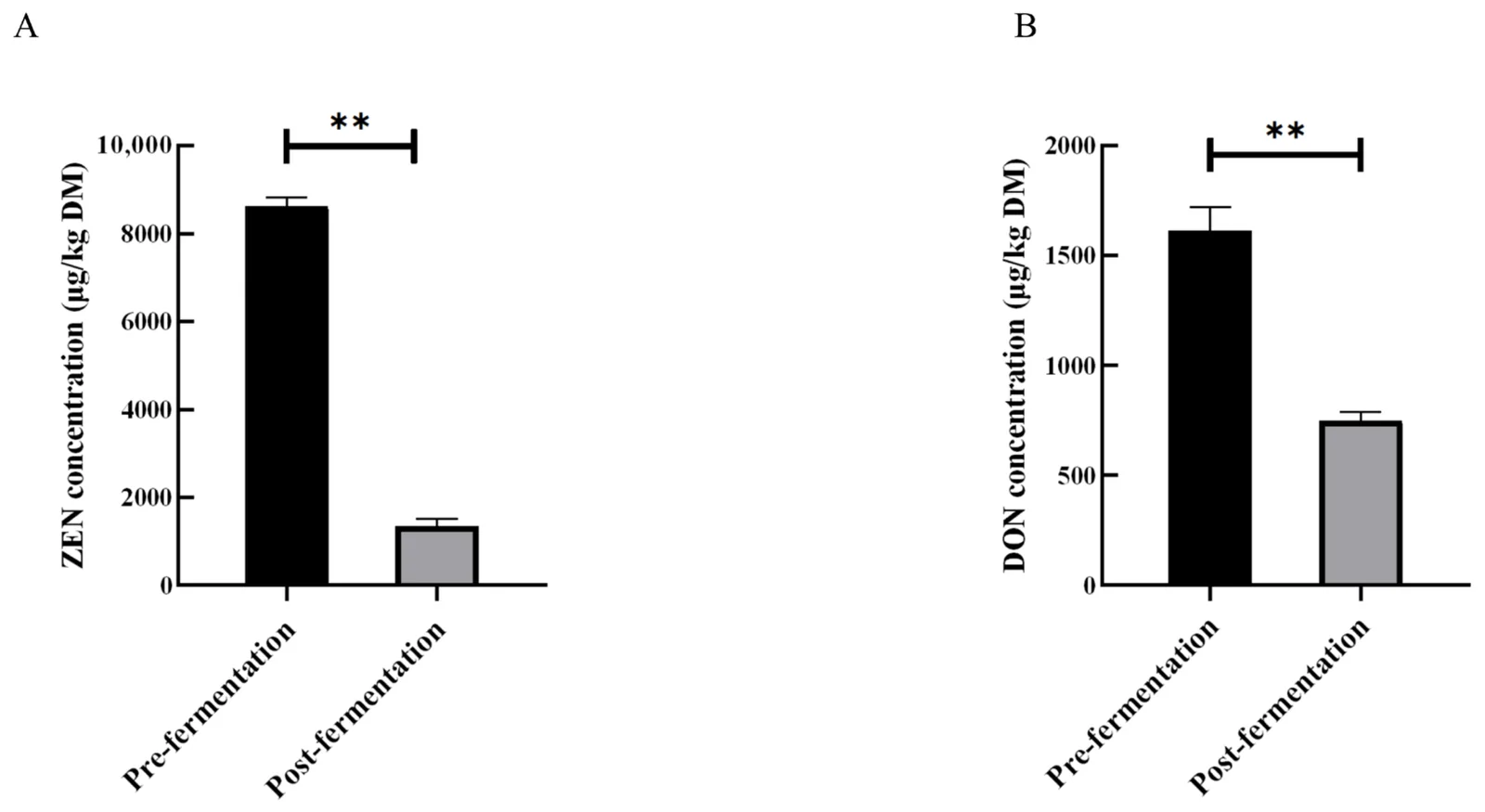
Changes in mycotoxin concentrations in the SFVS-based substrate mixture under the optimized fermentation conditions. Concentrations of zearalenone (ZEN; (**A**)) and deoxynivalenol (DON; (**B**)) before and after fermentation. Data are presented as means ± SEM (*n* = 3 independent fermentation replicates). ** indicates a significant difference between pre- and post-fermentation values (*p* < 0.01).

Under the optimal fermentation conditions, the ZEN and DON concentrations in the substrate mixture decreased from 8622.43 and 1612.74 μg/kg to 1349.82 and 750.21 μg/kg, respectively, corresponding to percentage reductions of 84.35% and 53.48% (*p* < 0.01; Figure 2A,B).

### 3.2. Effects of Mixed-Strain Solid-State Fermentation on the Nutrient Composition, Apparent Metabolizable Energy, and In Vitro Digestibility of the SFVS-Based Substrate Mixture

Table 3 and Table 4 present the nutrient composition and amino acid profile of the SFVS-based substrate mixture, respectively. Compared with the pre-fermentation sample, the fermented sample had lower pH, moisture, and CF contents but higher CP content and a higher total measured amino acid content (*p* < 0.05), whereas EE and GE did not differ (*p* > 0.05). AME did not differ between fermented and unfermented substrate mixtures (*p* > 0.05; Table 3). Fermentation also increased the in vitro digestibilities of DM, CP, and CF (*p* < 0.05), with CP digestibility increasing from 2.44% to 17.67% (Table 5).

### 3.3. Effects of Fermentation Status and Dietary Inclusion Level of the SFVS-Based Substrate Mixture on Growth Performance and Relative Organ Weights of Yellow-Feathered Broilers

Interactions between fermentation status and inclusion level were detected for several growth performance variables (*p* < 0.05; Table 6). Compared with CON and the corresponding unfermented treatments, both 3F and 5F had higher BW on days 21 and 42 and higher overall ADG, together with lower overall FCR (*p* < 0.05). The 3F had higher BW on day 42, overall ADG, and average daily feed intake (ADFI) than the 5F (*p* < 0.05), whereas BW on day 21 and overall FCR did not differ between the two groups (*p* > 0.05). In addition, compared with CON, 7F had lower ADG during days 1–21 (*p* < 0.05) but similar overall ADG (*p* > 0.05); and 7WF had higher overall FCR (*p* < 0.05). Effect-size estimates for overall growth performance further showed that, compared with CON, the mean differences in overall ADG were 2.103 and 1.232 g/d in the 3F and 5F, respectively. The corresponding mean differences in overall FCR were −0.130 and −0.138, respectively, whereas the mean difference in overall FCR in the 7WF was 0.054 (Table S5).

For relative organ weights, fermentation status affected relative heart and spleen weights (*p* < 0.05), with higher relative heart weights and lower relative spleen weight in the unfermented treatments. Inclusion level also affected relative spleen weight (*p* = 0.031). An interaction between fermentation status and inclusion level was detected for relative kidney weight (*p* = 0.020), although pairwise comparisons among the individual treatment combinations were not significant. Compared with CON, all unfermented treatments had higher relative heart weights, whereas 7F had lower relative liver weight and 7WF had lower relative spleen weight (*p* < 0.05; Table 7).

### 3.4. Effects of Fermentation Status and Dietary Inclusion Level of the SFVS-Based Substrate Mixture on Breast Meat Quality of Yellow-Feathered Broilers

Treatment-related differences in breast meat quality were mainly evident at 48 h postmortem and in cooking loss and texture-related traits. Except for 3F, all other dietary treatments containing the substrate mixture had higher breast muscle pH and lower a* values than CON at 48 h postmortem (*p* < 0.05; Table 8). Cooking loss, shear force, hardness, gumminess, and chewiness were affected by the fermentation status × inclusion level interaction (*p* < 0.05). Cooking loss was higher in 5WF and 7WF than in the corresponding fermented treatments and CON. Compared with CON, 7WF had lower shear force (*p* < 0.05; Table 9).

### 3.5. Effects of Fermentation Status and Dietary Inclusion Level of the SFVS-Based Substrate Mixture on Serum Antioxidant Indices and Hepatic Enzyme Activities of Yellow-Feathered Broilers

At the same inclusion level, the unfermented treatments had higher MDA concentrations but lower T-SOD and CAT activities than the corresponding fermented treatments; GSH-Px activity was also lower in the unfermented treatments at the 5% and 7% inclusion levels (*p* < 0.05). ALT and ALP activities were higher in the unfermented treatments than in the corresponding fermented treatments, and ALT, AST, and ALP activities were higher in 7WF than in CON (*p* < 0.05). Compared with 5F, CAT and ALT activities were higher in 3F, whereas ALP activity was lower (*p* < 0.05). MDA concentrations and T-SOD and GSH-Px activities did not differ between the two groups (*p* > 0.05). Fermentation status × inclusion level interactions were detected for all variables except AST (*p* < 0.05; Table 10).

### 3.6. Effects of Fermentation Status and Dietary Inclusion Level of the SFVS-Based Substrate Mixture on Liver and Jejunal Histomorphology of Yellow-Feathered Broilers

**Figure 3:**
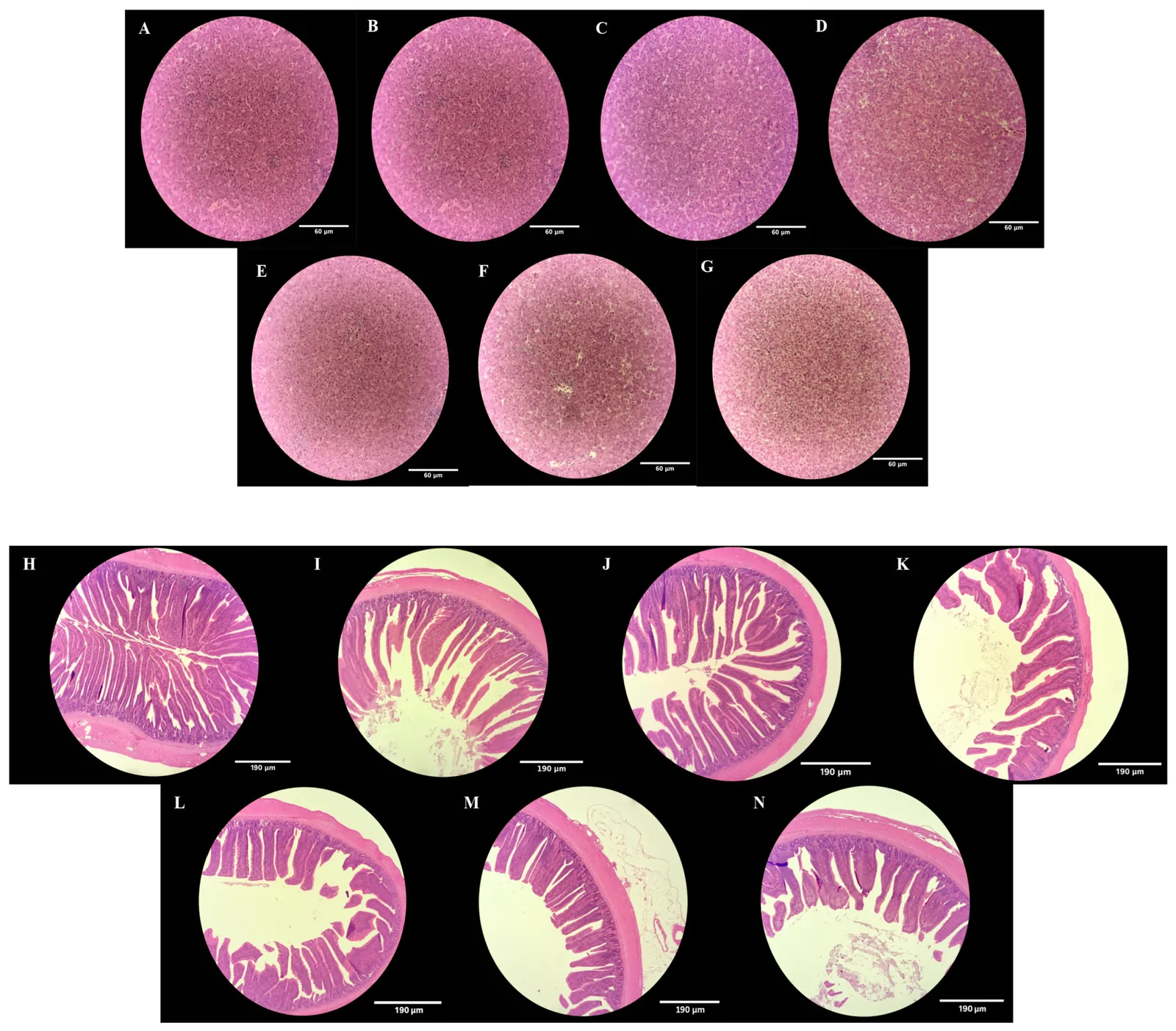
Effects of dietary treatments containing the fermented or unfermented SFVS-based substrate mixture on liver and jejunal histomorphology of yellow-feathered broilers. Representative hematoxylin and eosin-stained sections of the liver (**A**–**G**) and jejunum (**H**–**N**) (*n* = 6). In H&E-stained sections, nuclei appear blue-purple, whereas the cytoplasm and extracellular matrix appear pink. (**A**) and (**H**), CON; (**B**) and (**I**), 3F; (**C**) and (**J**), 5F; (**D**) and (**K**), 7F; (**E**) and (**L**), 3WF; (**F**) and (**M**), 5WF; and (**G**) and (**N**), 7WF. Scale bars = 60 μm for the liver sections (**A**–**G**) and 190 μm for the jejunal sections (**H**–**N**). CON = basal diet control; F and WF denote diets containing the fermented and unfermented substrate mixtures, respectively; numerals 3, 5, and 7 denote dietary inclusion levels of the substrate mixture (%).

H&E-stained liver sections from each treatment are shown in Figure 3A–G. CON and the groups receiving the fermented substrate mixture exhibited intact hepatic lobular architecture and regularly arranged hepatic cords, whereas the diets containing the unfermented substrate mixture showed histological changes characterized by disorganized hepatic cords, hepatocellular vacuolation, and inflammatory cell infiltration. All jejunal morphometric indices were affected by the fermentation status × inclusion level interaction (*p* < 0.001). At the same inclusion level, VH and VH:CD ratio were higher in each fermented treatment than in the corresponding unfermented treatment (*p* < 0.05). Compared with CON, VH was higher in 3F and 5F but lower in 7F and all unfermented treatments (*p* < 0.05). In addition, both VH and CD were higher in 3F than in 5F, whereas the VH:CD ratio was lower in 3F (*p* < 0.05; Figure 3H–N and Table 11).

### 3.7. Effects of Fermentation Status and Dietary Inclusion Level of the SFVS-Based Substrate Mixture on the Relative mRNA Expression of Jejunal Homeostasis-Related Genes in Yellow-Feathered Broilers

**Figure 4:**
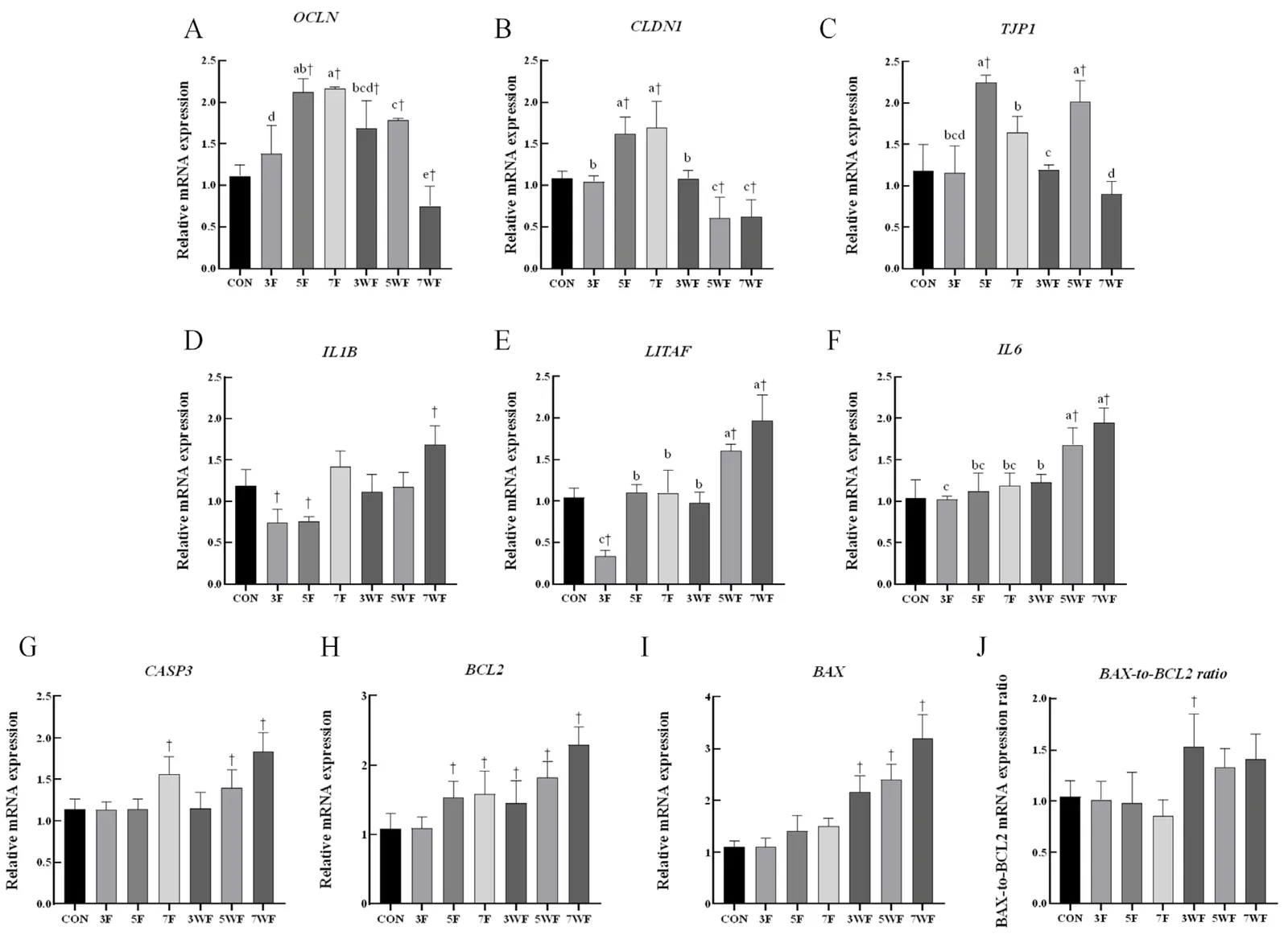
Effects of dietary treatments containing the SFVS-based substrate mixture on the relative mRNA expression of jejunal homeostasis-related genes in yellow-feathered broilers. Relative mRNA expression of occludin (*OCLN*; (**A**)), claudin-1 (*CLDN1*; (**B**)), tight junction protein 1 (*TJP1*; (**C**)), interleukin 1 beta (*IL1B*; (**D**)), lipopolysaccharide-induced TNF factor (*LITAF*; (**E**)), interleukin 6 (*IL6*; (**F**)), caspase 3 (*CASP3*; (**G**)), BCL2 apoptosis regulator (*BCL2*; (**H**)), and BCL2-associated X apoptosis regulator (*BAX*; (**I**)), as well as the *BAX*-to-*BCL2* mRNA expression ratio (**J**). Data are presented as means ± SEM (*n* = 6). When the fermentation status × inclusion level interaction was significant, treatment combinations not sharing a lowercase letter differed (Tukey- or Holm-adjusted *p* < 0.05). † Indicates a difference from CON (Dunnett- or Holm-adjusted *p* < 0.05). CON = basal diet control; SFVS = spent *Flammulina velutipes* substrate; F and WF denote diets containing the fermented and unfermented substrate mixtures, respectively; numerals 3, 5, and 7 denote dietary inclusion levels of the substrate mixture (%).

The relative mRNA expression levels of *IL1B* and *LITAF* were lower in 3F than in CON (*p* < 0.05). Compared with the corresponding unfermented treatment, 7F had higher jejunal mRNA expression of *CLDN1*, *OCLN*, and *TJP1* but lower *IL6* expression (*p* < 0.05). *BAX* mRNA expression was affected by fermentation status (*p* < 0.001) and inclusion level (*p* = 0.002), but not by their interaction (*p* = 0.053). *IL1B*, *LITAF*, *IL6*, *CASP3*, and *BAX* mRNA expression levels were higher in 7WF than in CON (*p* < 0.05), whereas the *BAX*-to-*BCL2* mRNA expression ratio did not differ between any fermented treatment and CON (*p* > 0.05; Figure 4A–J).

### 3.8. Effects of Fermentation Status and Dietary Inclusion Level of the SFVS-Based Substrate Mixture on the Relative mRNA Expression of Pancreatic Digestive Enzyme-Related Genes in Yellow-Feathered Broilers

**Figure 5:**
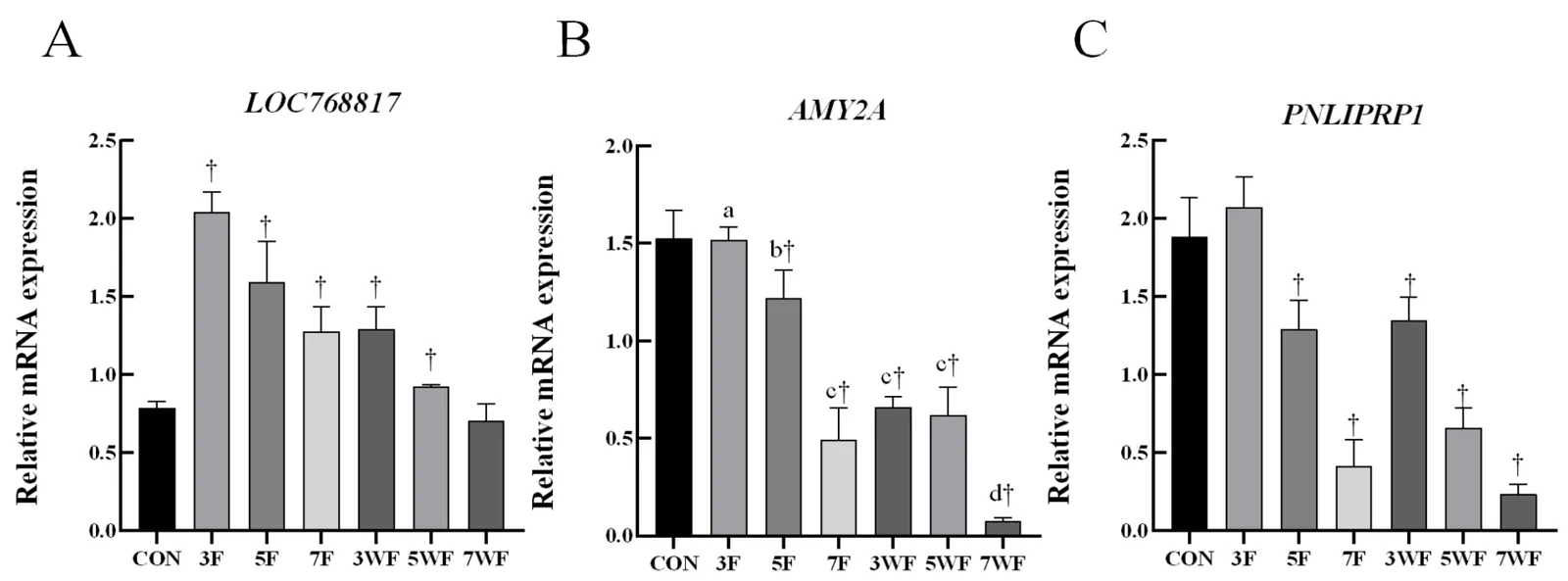
Effects of dietary treatments containing the SFVS-based substrate mixture on the relative mRNA expression of pancreatic digestive enzyme-related genes in yellow-feathered broilers. Relative mRNA expression of membrane metalloendopeptidase-like 1 (*LOC768817*; (**A**)), alpha-amylase 2A (*AMY2A*; (**B**)), and pancreatic lipase-related protein 1 (*PNLIPRP1*; (**C**)). Data are presented as means ± SEM (*n* = 6). When the fermentation status × inclusion level interaction was significant, treatment combinations not sharing a lowercase letter differed (Tukey- or Holm-adjusted *p* < 0.05). † Indicates a difference from CON (Dunnett- or Holm-adjusted *p* < 0.05). CON = basal diet control; SFVS = spent *Flammulina velutipes* substrate; F and WF denote diets containing the fermented and unfermented substrate mixtures, respectively; numerals 3, 5, and 7 denote dietary inclusion levels of the substrate mixture (%).

The mRNA expression of pancreatic digestive enzyme-related genes varied with fermentation status and inclusion level. At the same inclusion level, *AMY2A* mRNA expression was higher in all fermented treatments than in the corresponding unfermented treatments (*p* < 0.05). *PNLIPRP1* mRNA expression was affected by fermentation status and inclusion level (both *p* < 0.001), but not by their interaction (*p* = 0.153), with overall higher expression in the fermented treatments than in the unfermented treatments. *LOC768817* mRNA expression was higher in all fermented treatments and in 3WF and 5WF than in CON; except for 3F, the mRNA expression levels of *AMY2A* and *PNLIPRP1* were lower in all other SFVS treatments than in CON (*p* < 0.05; Figure 5A–C).

### 3.9. Effects of Dietary Treatments Containing the SFVS-Based Substrate Mixture on the Cecal Microbiota and Microbiota–Host Phenotype Associations in Yellow-Feathered Broilers

**Figure 6:**
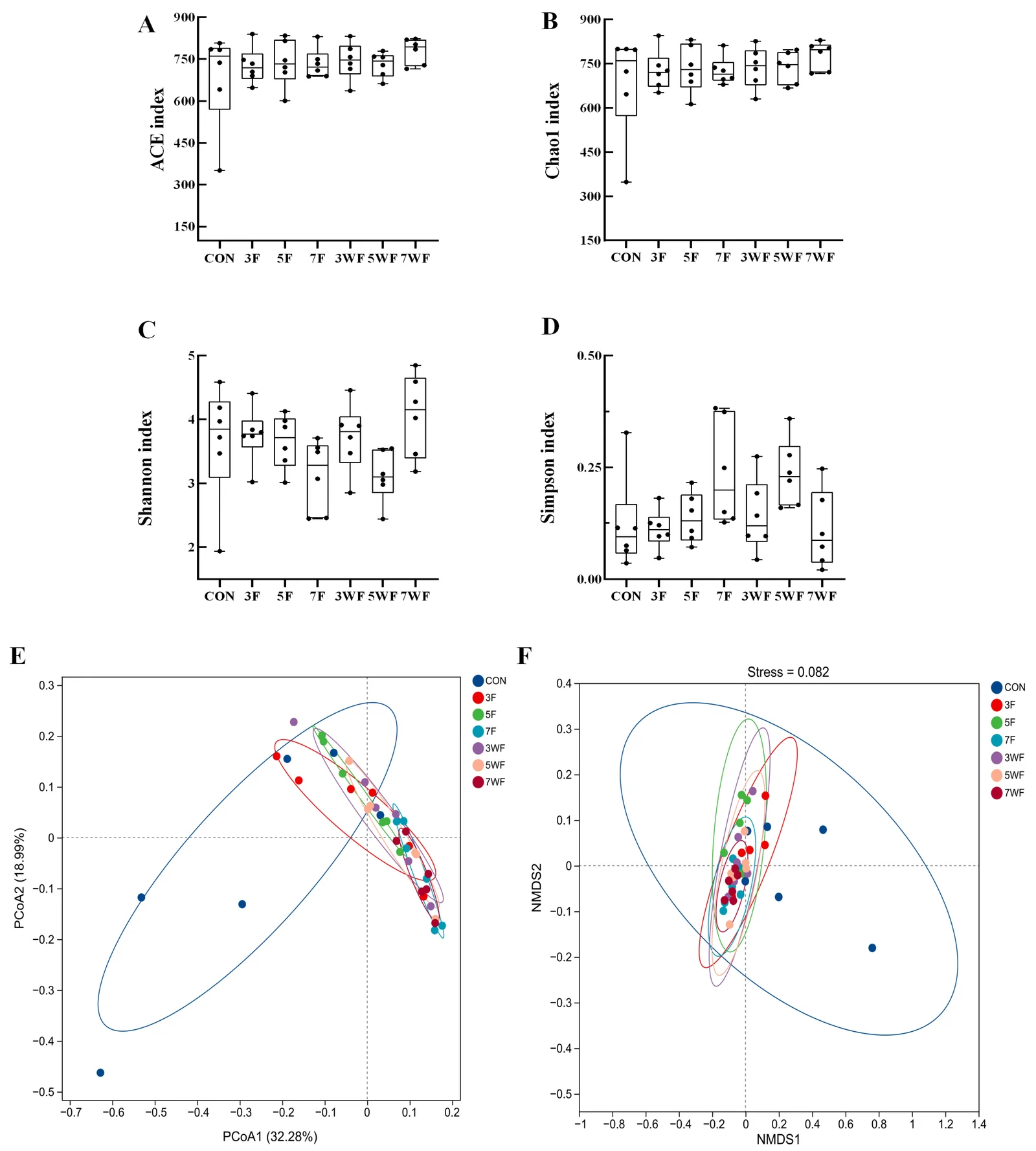
Effects of dietary treatments containing the SFVS-based substrate mixture on cecal microbial α-diversity and community structure in yellow-feathered broilers. Abundance-based coverage estimator (ACE; (**A**)), Chao1 index (**B**), Shannon index (**C**), and Simpson index (**D**), together with principal coordinates analysis (PCoA; (**E**)) and non-metric multidimensional scaling (NMDS; (**F**)) of the cecal bacterial community based on Bray–Curtis dissimilarities (*n* = 6). Each dot represents an individual sample. The ellipses in panels (**E**,**F**) indicate the distribution of samples within each treatment group. CON = basal diet control; F and WF denote diets containing the fermented and unfermented substrate mixtures, respectively; numerals 3, 5, and 7 denote dietary inclusion levels of the substrate mixture (%).

For the abundance-based coverage estimator (ACE) and the Chao1, Shannon, and Simpson indices, none of the dietary treatments containing the SFVS-based substrate mixture differed significantly from CON (*p* > 0.05; Figure 6A–D). PERMANOVA detected an overall effect of dietary treatment on cecal microbial community structure (pseudo-*F* = 4.95, *R*^2^ = 0.459, *p* = 0.002). In the ordinations generated by principal coordinates analysis (PCoA) and nonmetric multidimensional scaling (NMDS), the treatments containing the fermented substrate mixture were generally positioned closer to CON, whereas the unfermented treatments showed greater separation (Figure 6E,F).

**Figure 7:**
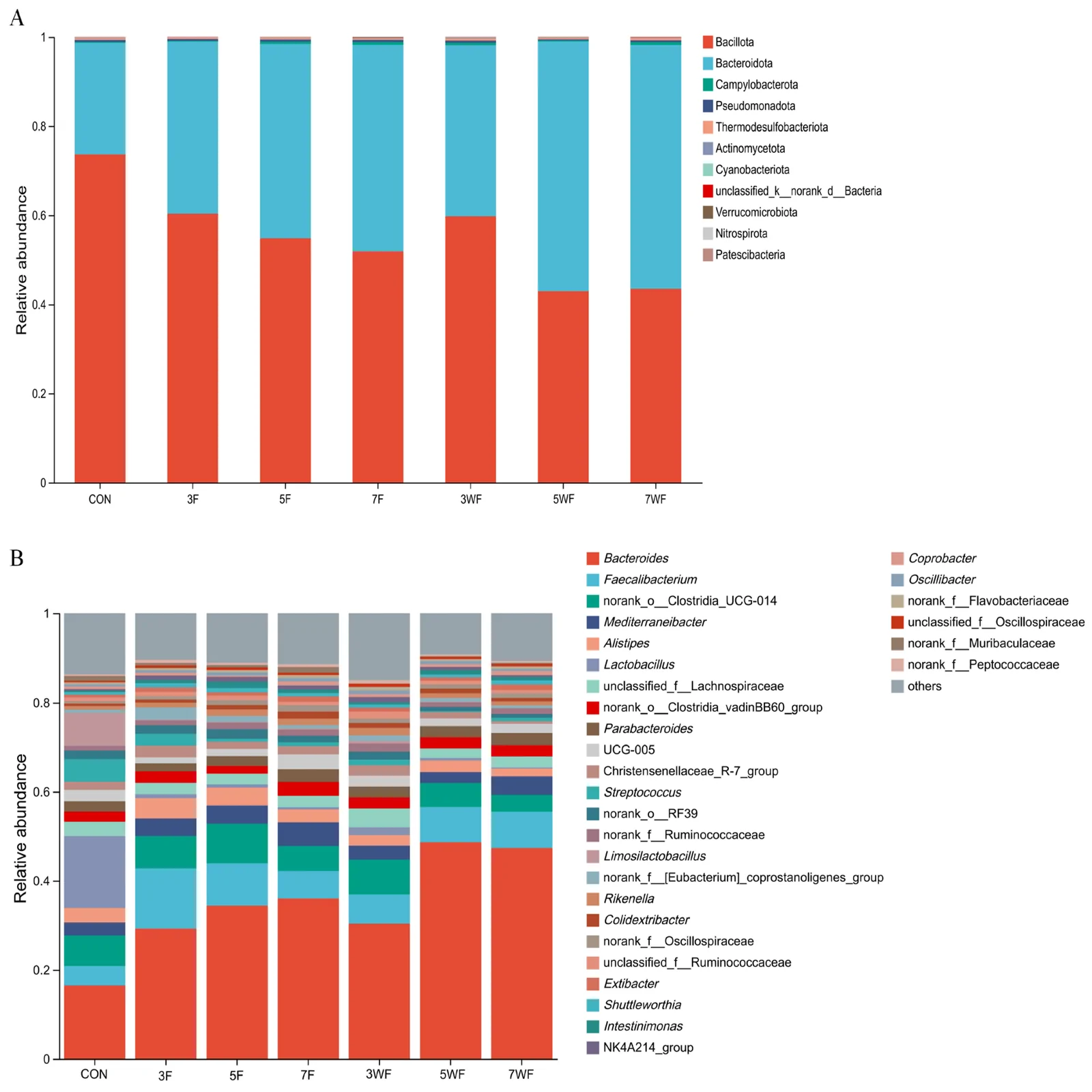
Effects of dietary treatments containing the SFVS-based substrate mixture on cecal microbial community composition in yellow-feathered broilers. Relative abundances of the predominant bacteria at the phylum (**A**) and genus (**B**) levels in cecal contents (*n* = 6). CON = basal diet control; F and WF denote diets containing the fermented and unfermented substrate mixtures, respectively; numerals 3, 5, and 7 denote dietary inclusion levels of the substrate mixture (%).

Phylum- and genus-level microbial community profiles are presented in Figure 7A and Figure 7B, respectively. As dietary level of the SFVS-based substrate mixture inclusion increased, the relative abundance of Bacillota declined overall, whereas that of Bacteroidota generally increased. At the genus level, the relative-abundance profiles showed generally higher relative abundances of *Bacteroides* and *Faecalibacterium* in dietary treatments containing the SFVS-based substrate mixture than in CON, with this pattern being more pronounced for *Faecalibacterium* in treatments containing the fermented SFVS-based substrate mixture.

**Figure 8:**
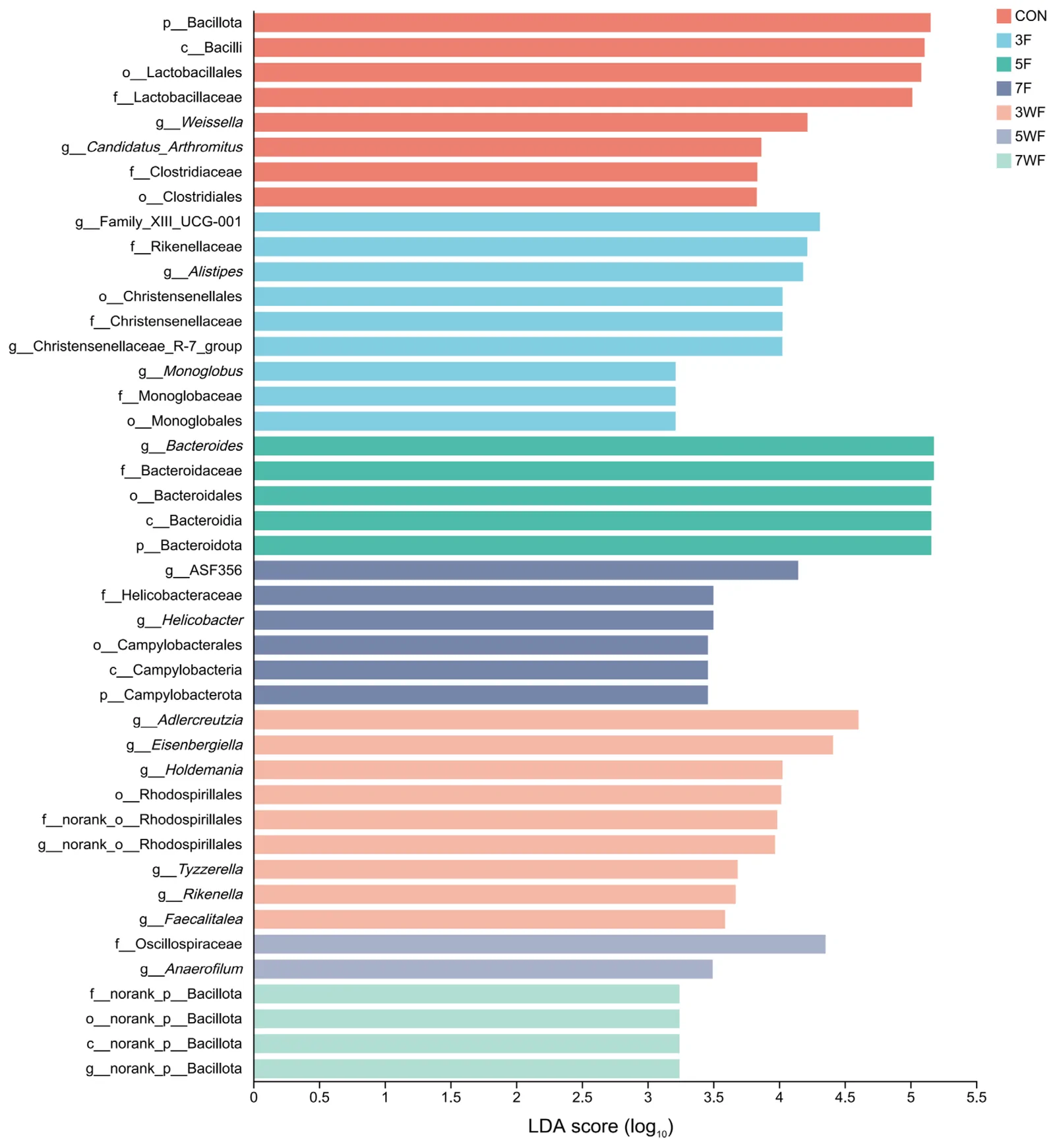
Effects of dietary treatments containing the SFVS-based substrate mixture on differentially abundant cecal bacterial taxa in yellow-feathered broilers. Linear discriminant analysis (LDA) score distribution of differentially abundant bacterial taxa identified by LEfSe. An LDA score ≥ 2.0 was used as the threshold for identifying differentially abundant taxa. CON = basal diet control; F and WF denote diets containing the fermented and unfermented substrate mixtures, respectively; numerals 3, 5, and 7 denote dietary inclusion levels of the substrate mixture (%).

Analysis using linear discriminant analysis effect size (LEfSe) indicated distinct microbial enrichment patterns among treatments. Differentially enriched taxa in CON included Lactobacillaceae and *Weissella*; 3F was characterized by *Alistipes*, Christensenellaceae_R-7_group, and *Monoglobus*; 5F showed enrichment of *Bacteroides* and other Bacteroidota-associated taxa; and Campylobacterota and *Helicobacter* were the major differentially enriched taxa in 7F. Differentially enriched taxa in the unfermented substrate mixture treatment groups mainly included *Adlercreutzia*, *Eisenbergiella*, *Holdemania*, and *Anaerofilum*, although the composition of biomarker taxa differed among the different dietary inclusion levels of unfermented substrate mixture (linear discriminant analysis (LDA) score ≥ 2.0; Figure 8 and Figure S1).

**Figure 9:**
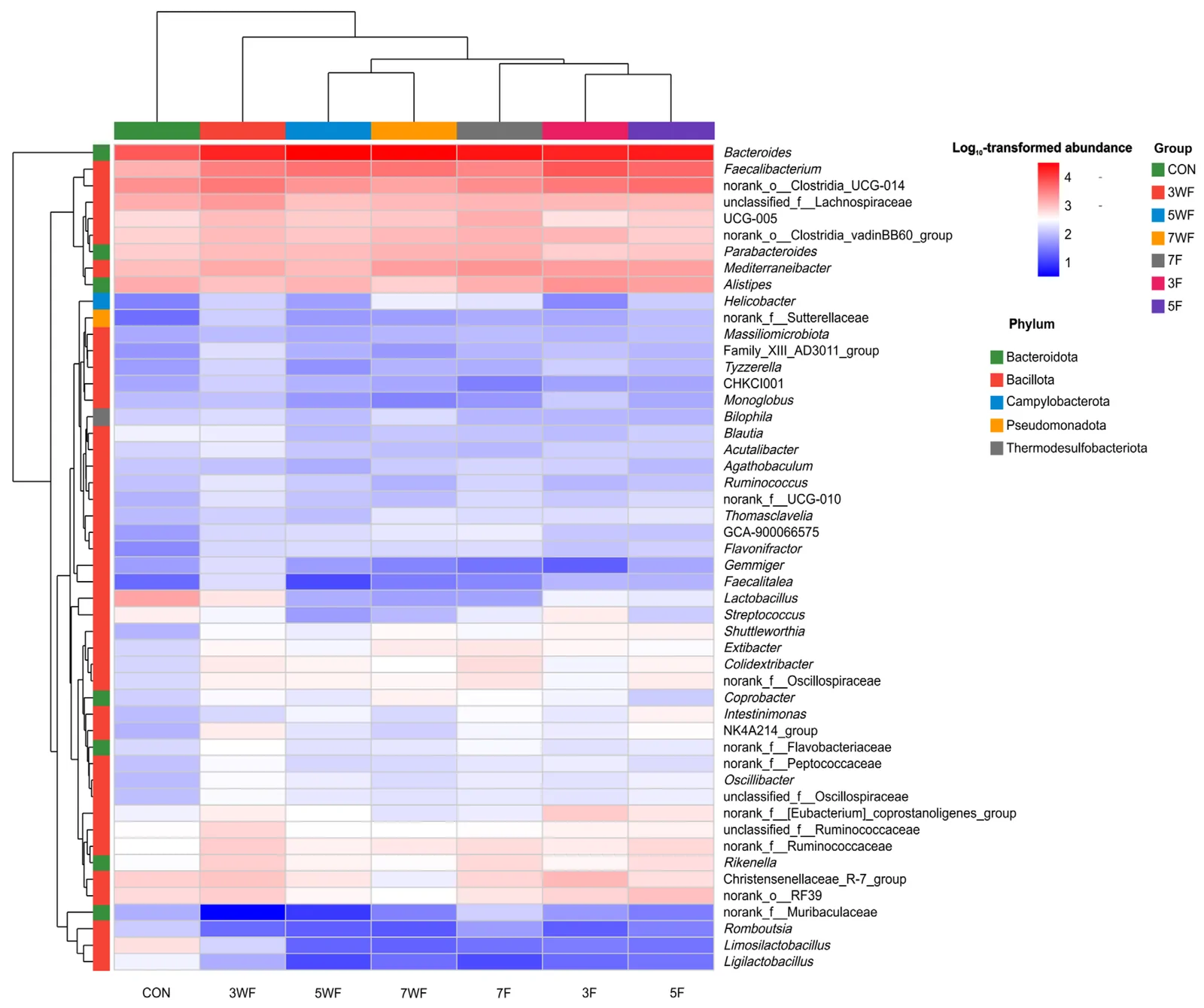
Effects of dietary inclusion of the fermented or unfermented SFVS-based substrate mixture on cecal microbial community composition in yellow-feathered broilers. Hierarchical clustering heatmap of log_10_-transformed bacterial abundances at the genus level. CON = basal diet control; F and WF denote diets containing the fermented and unfermented substrate mixtures, respectively; numerals 3, 5, and 7 denote dietary inclusion levels of the substrate mixture (%).

Genus-level clustering in the heatmap revealed differences in microbial community composition among dietary treatments (Figure 9). After Benjamini–Hochberg false discovery rate (FDR) correction across all 84 genus–phenotype correlations, only the relative abundance of *Alistipes* was significantly negatively correlated with breast muscle chewiness (Spearman’s ρ = −0.511, q = 0.0454), whereas the remaining correlations did not reach statistical significance after correction (Figure S2).

## 4. Discussion

Given that both ZEN and DON were detected in the substrate mixture used in the present study, reducing their detectable concentrations represents an important aspect of evaluating the effectiveness of the fermentation treatment. Microbe-mediated mycotoxin control generally exhibits a certain degree of selectivity toward target toxins and may reduce detectable toxin levels through multiple mechanisms [20]. A consortium of *Bacillus subtilis*, *Lactobacillus casei*, and *Candida utilis* degraded aflatoxin B1 (AFB_1_) and ZEN more effectively than *B. subtilis* alone [21]. In the present study, the detectable concentrations of ZEN and DON in the substrate mixture also decreased concurrently after mixed-strain fermentation. After the optimal mixed-strain inoculum ratio was determined, the combined reduction in ZEN and DON concentrations was closely associated with an appropriate combination of moisture content, total inoculum level, and fermentation time. However, the L9 (3^4^) orthogonal design did not permit independent estimation of interactions among the strain inoculation-ratio factors, and the formal experiment did not include systematic single-strain and simplified-consortium controls, limiting the determination of the individual contributions and interactions of the strains.

Based on the measured ZEN and DON concentrations in fermented and unfermented substrate mixtures and their dietary inclusion levels, the estimated contributions of the fermented substrate mixture to dietary ZEN and DON concentrations at inclusion levels of 3–7% were 40.49–94.49 and 22.51–52.51 μg/kg, respectively, compared with 258.67–603.57 and 48.38–112.89 μg/kg for the unfermented substrate mixture. In the context of the corresponding limits for other formula feeds specified in GB 13078-2017 (500 μg/kg for ZEN and 3000 μg/kg for DON), the estimated contribution of 7% unfermented substrate mixture to dietary ZEN was 603.57 μg/kg, suggesting that the potential mycotoxin burden associated with this treatment warrants attention [22]. However, these values represent only the theoretical contributions of the SFVS-based substrate mixtures to dietary mycotoxin levels, rather than the measured concentrations of ZEN and DON in the complete experimental diets. Because other feed ingredients may also contribute mycotoxins, these estimates are insufficient to determine actual dietary mycotoxin exposure or establish whether the complete diets comply with feed hygiene standards.

Comparisons of the nutritional composition and in vitro digestibility of the substrate mixture before and after fermentation indicated that mixed-strain SSF may have improved its nutritional quality and in vitro digestibility. It should be noted that the increases in CP concentration and the total concentration of the measured amino acids after fermentation do not necessarily indicate increases in the absolute amounts of these nutrients, as dry matter losses during fermentation may contribute to their relative enrichment in the fermented product. Microbial biomass protein accumulation and the enzymatic breakdown of complex fiber fractions and macromolecular nutrients may also enhance nutrient release and utilization [23]. Fermentation significantly increased the in vitro digestibility of DM, CP, and CF in the substrate mixture; however, AME did not increase significantly. This discrepancy may reflect differences in the aspects of nutrient utilization assessed by in vitro digestibility and AME. In vitro digestibility primarily reflects the extent to which specific nutrient fractions are digested under simulated digestive conditions, whereas AME reflects the animal’s overall utilization of energy from the feed ingredient. Although in vitro CP digestibility increased from 2.44% to 17.67%, the value remained low in absolute terms, and neither EE nor GE changed significantly after fermentation. The improvement in the in vitro digestibility of an individual nutrient fraction may therefore have been insufficient to produce a significant increase in AME.

The feeding value of the substrate mixture in broiler diets depends on processing method and inclusion level. Previous studies have reported that fermentation of plant-derived feed ingredients can improve broiler performance, although the specific responses vary depending on the feed substrate [24,25]. In the present study, the dietary treatments containing the fermented substrate mixture generally showed better growth performance and feed conversion efficiency than the corresponding unfermented treatments, but these advantages did not increase progressively with increasing inclusion level. Although 3F had higher BW on day 42, overall ADG, and overall ADFI than the 5F, overall FCR did not differ between the two groups. Compared with the CON, both 7F and 7WF showed lower ADG during the starter phase and lower pancreatic mRNA expression of *AMY2A* and *PNLIPRP1*, whereas ADG during the grower phase was higher in both groups. Neither group differed significantly from the CON in ADG over days 1–42, suggesting that improved in vitro digestibility does not necessarily translate into a significant improvement in overall growth performance. In addition, differences in dietary ingredient proportions, calculated lysine levels, and dietary fiber characteristics among treatments may also have influenced broiler growth performance and feed efficiency. Dietary fiber characteristics can influence nutrient utilization efficiency and growth performance in broilers [26]. At the higher substrate-mixture inclusion level, structural fiber, dietary bulk, and other potential residual components of the substrate mixture may be associated with the observed responses.

Regarding breast meat quality, the dietary treatments containing the unfermented substrate mixture at higher inclusion levels exhibited greater cooking loss. Cooking loss can serve as an indicator of muscle water-holding capacity during heating, and water loss arising from muscle protein denaturation and tissue contraction is generally linked to increased cooking loss [27]. Notably, compared with CON, shear force was lower in 7WF, whereas hardness, gumminess, and chewiness did not differ between the two groups. Because shear force and TPA parameters characterize different dimensions of meat texture, they need not change in the same direction [28]. Given the higher cooking loss in 7WF, water loss during heating may influence the mechanical response of muscle tissue during compression [29]. By contrast, low and moderate dietary inclusion levels of the fermented substrate mixture did not adversely affect breast muscle water-holding capacity during heating or overall texture.

Mixed mycotoxin exposure can induce oxidative stress and alter hepatic metabolic responses in broilers [30]. In the present study, differences were observed between the dietary treatment groups containing the fermented and unfermented substrate mixtures in serum antioxidant indices, hepatic enzyme activities, and liver histology. Although the detectable concentrations of ZEN and DON were lower in the fermented substrate mixture, the differences in these physiological indices between treatment groups cannot be directly attributed to reduced mycotoxin exposure in broilers. Changes in the nutritional characteristics and in vitro digestibility of the substrate mixture following fermentation, and microbial metabolites that may be produced during fermentation may also have contributed to these physiological responses.

As an inhibitor of protein synthesis, DON can directly compromise intestinal epithelial barrier integrity and cause villus shortening; ZEN can induce oxidative stress in the intestinal epithelium and disrupt tight junction homeostasis [31,32,33]. In the present study, 3F had greater VH and CD but a lower VH:CD ratio than 5F. These findings suggest that the two inclusion levels had differential effects on individual jejunal morphometric indices, and that the overall superiority of either level cannot be established on the basis of a single index alone. Compared with 7WF, 7F showed higher jejunal mRNA expression of *CLDN1*, *OCLN*, and *TJP1*, suggesting an association between fermentation and higher expression of tight-junction-related genes at the higher inclusion level. In addition to changes in mycotoxin levels, fermentation-associated acidification may also contribute to the modulation of the intestinal environment [34]. The pH of the SFVS-based substrate mixture decreased from 5.89 to 5.65 after fermentation, which may reflect the accumulation of acidic metabolites. Because only the mRNA expression of tight junction-related genes was measured and organic acids such as lactic, acetic, and butyric acids were not quantified, the specific roles of the corresponding tight junction proteins and fermentation-associated organic acids in these responses require further investigation.

The structure and extent of degradation of dietary fiber can alter substrate availability to cecal microorganisms [35,36]; DON or ZEN exposure can also alter cecal microbial composition in broiler chickens [37,38]. The cecal microbial profiles of the fermented treatments were generally more similar to CON, whereas the unfermented treatments deviated more markedly from CON, suggesting that the response of the cecal microbiota profiles of the dietary treatments containing the fermented substrate mixture primarily involved restructuring of community composition rather than overall changes in microbial richness and diversity.

After Benjamini–Hochberg false discovery rate (FDR) correction, the only significant association identified in the present study was a negative correlation between the relative abundance of *Alistipes* and breast muscle chewiness (Spearman’s ρ = −0.511, q = 0.0454). Changes in the abundance of *Alistipes* are associated with growth and metabolic traits in chickens [39]; *Bacteroides* has the potential to utilize complex polysaccharides and may participate in short-chain fatty acid metabolism [40]; *Faecalibacterium* has butyrate-producing potential and may contribute to the maintenance of intestinal homeostasis [41]; whereas *Helicobacter pullorum* can colonize the ceca of broilers and is associated with intestinal health status in broilers [42]. It should be noted that correlation analysis reflects statistical associations between the relative abundance of bacterial genera and host phenotypes but does not establish that changes in the microbiota caused changes in host phenotypes; dietary treatments may have independently influenced both microbiota composition and host phenotypes. Given that the present study relied mainly on 16S rRNA gene sequencing and correlation analysis, future studies could incorporate measurements of short-chain fatty acids, bile acids, and toxin metabolites to further investigate the potential links between gut microbiota and host phenotypes.

Overall, this study evaluated the feeding value of the fermented SFVS-based substrate mixture from multiple perspectives, including detectable mycotoxin levels, nutritional characteristics, growth performance, histological indices, and cecal microbiota. However, interpretation of its practical feeding value and underlying mechanisms remains subject to limitations in the experimental design and analytical methods. With respect to fermentation design and mycotoxin assessment, the detectable concentrations of ZEN and DON before and after fermentation were determined only by ELISA. Potential matrix effects were not assessed, and neither desorption assays nor liquid chromatography–tandem mass spectrometry (LC–MS/MS) analyses were performed. Consequently, the current results do not allow adsorption to be distinguished from transformation or degradation, nor do they permit identification of the associated products. In addition, dry matter recovery before and after fermentation was not determined; therefore, reductions in ZEN and DON calculated from concentration changes cannot be directly equated with reductions in total toxin mass. Regarding feeding value and host responses, dietary fiber characteristics may influence nutrient utilization in broilers. Because the fiber composition and bulk density of the complete experimental diets were not determined and relevant residual components were not directly measured, the specific contributions of these factors to the growth responses observed at higher inclusion levels remain unclear. Furthermore, AME was determined using the force-feeding method in adult roosters (*n* = 4 per treatment) without correction for endogenous energy losses, and the in vitro digestibility method was not independently validated for high-fiber fermented SFVS-based substrate mixtures. Accordingly, these results are primarily suitable for comparing the apparent metabolizable energy and in vitro digestibility characteristics of fermented and unfermented samples under the same assay conditions and should not be directly extrapolated to the AME, in vivo digestibility, or nutrient utilization of the substrate mixture in young yellow-feathered broilers. Furthermore, the broiler feeding trial was not preceded by an a priori power analysis, and no study-wide correction for multiple testing across all animal-trial outcome variables was applied. Future studies could combine dry matter recovery measurements, desorption assays, and chromatographic–mass spectrometric analyses to clarify toxin mass balance, distinguish adsorption from transformation or degradation, identify potential transformation products, and evaluate their toxicological relevance. Validated methods for evaluating nutrient utilization in high-fiber fermented substrates could also help clarify the effects of fermentation on nutrient utilization in broilers.

## 5. Conclusions

In this study, a mixed-strain SSF system comprising *Saccharomyces cerevisiae*, *Bacillus coagulans*, *Clostridium butyricum*, and *Bacillus subtilis* was established and optimized. Fermentation reduced the detectable concentrations of ZEN and DON in the substrate mixture and improved its nutritional quality and in vitro digestibility. Compared with the corresponding unfermented treatments, dietary inclusion of the fermented substrate mixture at 3% or 5% promoted growth, improved feed utilization, and was associated with more favorable serum antioxidant status and jejunal morphology in yellow-feathered broilers. At these inclusion levels, the fermented substrate mixture did not adversely affect breast meat cooking loss or overall texture. In addition, the cecal microbial community structures in broilers receiving diets containing the fermented substrate mixture were generally more similar to CON. Increasing the inclusion level to 7% provided no additional overall feeding benefit and was accompanied by unfavorable changes in several measured outcomes.

## Figures and Tables

**Table 1:** Ingredient composition and nutrient levels of experimental diets for yellow-feathered broilers ^1^.

**Item**	**Starter Phase (Days 1–21)**						
	**CON**	**3F**	**5F**	**7F**	**3WF**	**5WF**	**7WF**
Ingredient composition, %							
Corn	55.25	52.46	50.70	48.54	52.25	50.30	48.00
Soybean meal	34.30	32.37	30.85	30.08	32.45	31.00	30.60
Corn gluten meal	2.05	3.41	4.50	4.99	3.54	4.70	5.00
Soybean oil	2.00	2.36	2.55	2.99	2.36	2.60	3.00
Unfermented substrate mixture	0	—	—	—	3.00	5.00	7.00
Fermented substrate mixture	0	3.00	5.00	7.00	—	—	—
Dicalcium phosphate	1.40	1.40	1.40	1.40	1.40	1.40	1.40
Premix ^2^	5.00	5.00	5.00	5.00	5.00	5.00	5.00
Total	100	100	100	100	100	100	100
Nutrient levels ^3^							
Crude protein (CP), %	21.23	21.23	21.23	21.21	21.23	21.23	21.20
Metabolizable energy (ME), kcal/kg	2921	2921	2921	2923	2921	2923	2923
Calcium (Ca), %	1.06	1.06	1.06	1.06	1.06	1.06	1.06
Total phosphorus (P), %	0.64	0.62	0.62	0.62	0.63	0.62	0.62
Methionine (Met), %	0.44	0.45	0.45	0.45	0.45	0.46	0.46
Lysine (Lys), %	1.28	1.25	1.22	1.21	1.25	1.23	1.22
**Item**	**Grower Phase (Days 22–42)**						
	**CON**	**3F**	**5F**	**7F**	**3WF**	**5WF**	**7WF**
Ingredient composition, %							
Corn	61.60	58.37	56.40	54.12	58.05	55.95	53.60
Soybean meal	27.50	26.58	25.44	25.04	26.83	25.84	25.40
Corn gluten meal	3.20	3.80	4.61	4.85	3.80	4.61	5.00
Soybean oil	1.70	2.25	2.55	2.99	2.32	2.60	3.00
Unfermented substrate mixture	0	—	—	—	3.00	5.00	7.00
Fermented substrate mixture	0	3.00	5.00	7.00	—	—	—
Dicalcium phosphate	1.00	1.00	1.00	1.00	1.00	1.00	1.00
Premix ^2^	5.00	5.00	5.00	5.00	5.00	5.00	5.00
Total	100	100	100	100	100	100	100
Nutrient levels ^3^							
Crude protein (CP), %	19.31	19.31	19.31	19.31	19.31	19.31	19.31
Metabolizable energy (ME), kcal/kg	2988	2985	2988	2988	2988	2985	2985
Calcium (Ca), %	0.97	0.97	0.97	0.97	0.97	0.97	0.97
Total phosphorus (P), %	0.52	0.52	0.51	0.51	0.52	0.51	0.51
Methionine (Met), %	0.43	0.43	0.43	0.43	0.43	0.43	0.44
Lysine (Lys), %	1.12	1.11	1.09	1.09	1.11	1.10	1.10

^1^ CON = basal diet control; F and WF denote fermented and unfermented substrate mixtures, respectively; numerals 3, 5, and 7 denote dietary inclusion levels (%). A dash (—) indicates that the corresponding substrate mixture was not included in the diet. ^2^ The premix provided the following per kilogram of diet: vitamin A, 7500 IU; vitamin D_3_, 1750 IU; vitamin E, 25 IU; vitamin K_3_, 1.40 mg; vitamin B_1_, 4.00 mg; vitamin B_2_, 6.25 mg; copper, 12.5 mg; iron, 165 mg; manganese, 90 mg; zinc, 92.5 mg; iodine, 1.00 mg; and selenium, 0.20 mg. ^3^ Nutrient levels were calculated from the diet formulation and were not analytically determined in the complete experimental diets.

**Table 2:** Orthogonal optimization of the inoculum ratio of four microbial strains for solid-state fermentation of an SFVS-based substrate mixture.

Run	Factor ^1^				Weighted Score ^2^
	A	B	C	D	
1	1	1	1	1	39.31 ± 0.13
2	1	2	2	2	31.79 ± 0.62
3	1	3	3	3	19.95 ± 0.05
4	2	1	2	3	58.61 ± 0.12
5	2	2	3	1	44.61 ± 0.38
6	2	3	1	2	36.49 ± 0.32
7	3	1	3	2	48.74 ± 0.15
8	3	2	1	3	47.40 ± 0.35
9	3	3	2	1	32.92 ± 0.10
*K*_1_	91.05	146.66	123.20	116.84	
*K*_2_	139.71	123.80	123.32	117.02	
*K*_3_	129.06	89.36	113.30	125.96	
*k*_1_	30.35	48.89	41.07	38.95	
*k*_2_	46.57	41.27	41.11	39.01	
*k*_3_	43.02	29.79	37.77	41.99	
Range (*R*)	16.22	19.10	3.34	3.04	
Factor ranking	B > A > C > D				
Optimal level	A_2_	B_1_	C_2_	D_3_	
Optimal combination	A_2_B_1_C_2_D_3_				

^1^ A = *Saccharomyces cerevisiae* JGSW0229; B = *Bacillus coagulans* Nzrj360; C = *Clostridium butyricum* CGMCC No. 8187; D = *Bacillus subtilis* CICC 22366. Levels 1, 2, and 3 denote relative inoculum proportions of 1, 2, and 3, respectively. ^2^ Weighted scores are presented as means ± SEM from 3 independent fermentation replicates. *K*_1_*–K*_3_ represent the sums of the weighted-score means at levels 1–3, respectively; *k*_1_*–k*_3_ represent the corresponding level means; *R* represents the range among *k*_1_*–k*_3_. The weighted score was calculated as follows: weighted score = 0.6 × percentage reduction in ZEN concentration + 0.4 × percentage reduction in DON concentration. SEM = standard error of the mean; ZEN = zearalenone; DON = deoxynivalenol.

**Table 3:** Nutrient composition and apparent metabolizable energy of the SFVS-based substrate mixture before and after mixed-strain solid-state fermentation ^1^.

Analyte	Before Fermentation	After Fermentation	*p*-Value
pH	5.89 ± 0.03	5.65 ± 0.03	<0.01
Moisture, %	47.34 ± 0.53	44.00 ± 0.97	<0.05
Crude protein (CP), %	8.45 ± 0.25	11.35 ± 0.29	<0.01
Ether extract (EE), %	2.30 ± 0.08	2.48 ± 0.07	0.62
Crude fiber (CF), %	15.40 ± 0.19	11.56 ± 0.07	<0.01
Gross energy (GE), kcal/kg	3944 ± 31	3929 ± 22	0.74
Apparent metabolizable energy (AME), kcal/kg	1004 ± 151	1128 ± 81	0.50

^1^ Values are presented as means ± SEM. Nutrient composition measurements were based on 3 independent replicates, whereas AME measurements were based on 4 replicates per treatment. Except for pH and moisture, all values are expressed on a dry matter basis.

**Table 4:** Amino acid composition of the SFVS-based substrate mixture before and after mixed-strain solid-state fermentation on a dry matter basis ^1^.

Amino Acid	Amino Acid Content		*p*-Value
	Before Fermentation, %	After Fermentation, %	
Aspartic acid (Asp)	1.24 ± 0.01	1.33 ± 0.02	<0.01
Threonine (Thr)	0.42 ± 0.01	0.47 ± 0.02	0.05
Serine (Ser)	0.36 ± 0.01	0.42 ± 0.01	<0.01
Glutamic acid (Glu)	1.56 ± 0.03	1.71 ± 0.02	<0.01
Glycine (Gly)	0.65 ± 0.01	0.81 ± 0.01	<0.01
Alanine (Ala)	0.60 ± 0.01	0.78 ± 0.02	<0.01
Cystine (Cys)	0.04 ± 0.02	0.04 ± 0.01	0.99
Valine (Val)	0.52 ± 0.01	0.59 ± 0.01	<0.05
Methionine (Met)	0.07 ± 0.01	0.08 ± 0.02	0.29
Isoleucine (Ile)	0.40 ± 0.03	0.46 ± 0.01	<0.05
Leucine (Leu)	0.68 ± 0.01	0.75 ± 0.01	<0.01
Tyrosine (Tyr)	0.21 ± 0.06	0.29 ± 0.01	0.34
Phenylalanine (Phe)	0.44 ± 0.01	0.48 ± 0.01	<0.01
Histidine (His)	0.26 ± 0.01	0.25 ± 0.01	0.29
Lysine (Lys)	0.49 ± 0.02	0.52 ± 0.01	0.08
Arginine (Arg)	0.53 ± 0.01	0.54 ± 0.01	0.48
Proline (Pro)	0.55 ± 0.01	0.67 ± 0.01	<0.01
Total measured amino acid content	9.02 ± 0.13	10.19 ± 0.11	<0.01

^1^ Values are reported as means ± SEM from 3 independent replicates.

**Table 5:** In vitro digestibility of dry matter, crude protein, and crude fiber in the SFVS-based substrate mixture before and after mixed-strain solid-state fermentation ^1^.

Analyte	Before Fermentation	After Fermentation	*p*-Value
Dry matter (DM), %	60.47 ± 1.07	69.00 ± 0.45	<0.05
Crude protein (CP), %	2.44 ± 0.70	17.67 ± 0.61	<0.01
Crude fiber (CF), %	31.31 ± 0.03	38.69 ± 0.10	<0.01

^1^ Values are reported as means ± SEM from 3 independent replicates.

**Table 6:** Effects of fermentation status and dietary inclusion level of the SFVS-based substrate mixture on growth performance of yellow-feathered broilers ^1^.

Variable	CON	Fermented Substrate Mixture			Unfermented Substrate Mixture			SEM ^2^	*p*-Value		
		3F	5F	7F	3WF	5WF	7WF		Fermentation Status	Inclusion Level	Fermentation Status × Inclusion Level
Initial BW, g	35.97	36.05	35.88	35.75	35.55	35.80	35.68	0.14	0.059	0.647	0.210
BW on day 21, g	243.06	292.17 ^a†^	273.67 ^a†^	213.96 ^b†^	232.41 ^b^	228.72 ^b^	210.18 ^b†^	5.73	<0.001	<0.001	<0.001
BW on day 42, g	1137.51	1225.91 ^a†^	1189.17 ^b†^	1138.60 ^c^	1140.47 ^c^	1137.20 ^c^	1129.81 ^c^	4.54	<0.001	<0.001	<0.001
ADG, days 1–21, g/d	9.86	12.20 ^a†^	11.32 ^a†^	8.49 ^b†^	9.37 ^b^	9.19 ^b^	8.31 ^b†^	0.27	<0.001	<0.001	<0.001
ADFI, days 1–21, g/d	31.49	32.71 ^a^	29.47 ^c†^	29.60 ^c†^	30.87 ^bc^	31.23 ^ab^	31.35 ^ab^	0.36	0.064	<0.001	<0.001
FCR, days 1–21, g:g	3.20	2.69 ^†^	2.61 ^†^	3.50	3.32	3.42	3.79 ^†^	0.11	<0.001	<0.001	0.063
ADG, days 22–42, g/d	42.59	44.46 ^†^	43.60	44.03 ^†^	43.24	43.26	43.79 ^†^	0.28	0.015	0.194	0.176
ADFI, days 22–42, g/d	99.38	101.32	99.98	100.72	100.05	99.62	101.44 ^†^	0.53	0.489	0.062	0.191
FCR, days 22–42, g:g	2.33	2.28	2.29	2.29	2.32	2.30	2.32	0.02	0.110	0.939	0.782
ADG, days 1–42, g/d	26.23	28.33 ^a†^	27.46 ^b†^	26.26 ^c^	26.31 ^c^	26.22 ^c^	26.05 ^c^	0.11	<0.001	<0.001	<0.001
ADFI, days 1–42, g/d	65.44	67.02 ^a†^	64.72 ^c^	65.16 ^bc^	65.46 ^bc^	65.43 ^bc^	66.39 ^ab^	0.31	0.616	0.003	<0.001
FCR, days 1–42, g:g	2.50	2.37 ^c†^	2.36 ^c†^	2.48 ^b^	2.49 ^b^	2.50 ^b^	2.55 ^a†^	0.01	<0.001	<0.001	0.010

^1^ Each treatment mean was derived from 6 replicate cages; CON = basal diet control; F and WF denote diets containing the fermented and unfermented substrate mixtures, respectively; numerals 3, 5, and 7 denote dietary inclusion levels (%); BW = body weight; ADG = average daily gain; ADFI = average daily feed intake; FCR = feed conversion ratio. ^2^ The six dietary treatments containing the fermented or unfermented substrate mixture were analyzed as a 2 × 3 factorial arrangement with fermentation status (fermented vs. unfermented), inclusion level (3%, 5%, and 7%), and their interaction as fixed effects; CON was excluded from the factorial analysis. SEM represents the pooled standard error of a treatment-combination mean derived from the residual mean square of the factorial model. Lowercase superscripts: Within a row, means among the six dietary treatment combinations lacking a common lowercase superscript differ significantly based on Tukey-adjusted comparisons (*p* < 0.05) when the fermentation status × inclusion level interaction was significant.^†^ Indicates a significant difference from CON based on Dunnett-adjusted comparisons (*p* < 0.05).

**Table 7:** Effects of fermentation status and dietary inclusion level of the SFVS-based substrate mixture on relative organ weights of yellow-feathered broilers ^1^.

Organ, g/kg BW	CON	Fermented Substrate Mixture			Unfermented Substrate Mixture			SEM ^2^	*p*-Value		
		3F	5F	7F	3WF	5WF	7WF		Fermentation Status	Inclusion Level	Fermentation Status × Inclusion Level
Heart	5.32	5.88	5.79	5.77	6.67 ^†^	7.18 ^†^	7.58 ^†^	0.20	<0.001	0.149	0.052
Liver	21.32	21.73	20.40	18.38 ^†^	21.75	21.79	22.36	0.99	0.052	0.405	0.116
Spleen	1.50	1.67	1.89	1.40	1.52	1.19	1.08 ^†^	0.20	0.033	0.031	0.502
Kidneys	8.37	8.29 ^a^	8.89 ^a^	7.69 ^a^	7.98 ^a^	8.15 ^a^	8.74 ^a^	0.31	0.997	0.434	0.020
Bursa of Fabricius	3.18	3.22	3.60	3.56	3.19	3.44	3.35	0.42	0.711	0.733	0.978

^1^ Each treatment mean was derived from 6 birds, with 1 representative bird selected from each replicate cage; CON = basal diet control; F and WF denote diets containing the fermented and unfermented substrate mixtures, respectively; numerals 3, 5, and 7 denote dietary inclusion levels (%); BW = body weight. ^2^ The six dietary treatments containing the fermented or unfermented substrate mixture were analyzed as a 2 × 3 factorial arrangement with fermentation status (fermented vs. unfermented), inclusion level (3%, 5%, and 7%), and their interaction as fixed effects; CON was excluded from the factorial analysis. SEM represents the pooled standard error of a treatment-combination mean derived from the residual mean square of the factorial model. Lowercase superscripts: Within a row, means among the six dietary treatment combinations lacking a common lowercase superscript differ when the fermentation status × inclusion level interaction was significant (Tukey- or Holm-adjusted *p* < 0.05). ^†^ Indicates a difference from CON (Dunnett- or Holm-adjusted *p* < 0.05).

**Table 8:** Effects of fermentation status and dietary inclusion level of the SFVS-based substrate mixture on breast muscle pH and color of yellow-feathered broilers ^1^.

Trait	Postmortem Time	CON	Fermented Substrate Mixture			Unfermented Substrate Mixture			SEM ^2^	*p*-Value		
			3F	5F	7F	3WF	5WF	7WF		Fermentation Status	Inclusion Level	Fermentation Status × Inclusion Level
pH	45 min	6.12	6.09	6.09	6.11	6.12	6.13	6.12	0.02	0.171	0.925	0.896
	24 h	5.88	5.89	5.87	5.84	5.84	5.83	5.86	0.02	0.183	0.553	0.126
	48 h	5.61	5.61 ^b^	5.76 ^a†^	5.73 ^ab†^	5.84 ^a†^	5.84 ^a†^	5.75 ^a†^	0.03	<0.001	0.045	0.009
Lightness (L*)	45 min	45.95	48.48 ^†^	46.65	48.56 ^†^	48.81 ^†^	48.72 ^†^	48.17	0.60	0.181	0.268	0.124
	24 h	53.16	53.60 ^a^	48.56 ^b†^	53.56 ^a^	51.84 ^a^	52.95 ^a^	53.06 ^a^	0.61	0.162	<0.001	<0.001
	48 h	55.00	55.20 ^a^	51.00 ^b†^	55.10 ^a^	54.00 ^ab^	55.00 ^a^	55.10 ^a^	0.79	0.156	0.031	0.007
Redness (a*)	45 min	0.19	0.13	0.36	0.21	0.10	−0.03	−0.09	0.22	0.183	0.898	0.698
	24 h	−0.33	−0.46	−0.89	−1.01	−0.99	−0.90	−0.87	0.18	0.385	0.457	0.165
	48 h	−0.65	−0.75 ^a^	−1.20 ^b†^	−1.35 ^b†^	−1.25 ^ab†^	−1.20 ^b†^	−1.15 ^ab†^	0.11	0.305	0.084	0.010
Yellowness (b*)	45 min	5.02	4.05 ^†^	4.22 ^†^	1.82 ^†^	2.97 ^†^	2.93 ^†^	1.84 ^†^	0.31	0.004	<0.001	0.098
	24 h	6.03	4.93 ^ab†^	5.33 ^ab†^	3.89 ^cd†^	3.56 ^d†^	4.61 ^bc†^	5.76 ^a†^	0.22	0.675	0.007	<0.001
	48 h	6.50	5.30 ^bc†^	5.80 ^ab†^	4.40 ^d†^	4.00 ^d†^	5.10 ^c†^	6.20 ^a^	0.12	0.505	<0.001	<0.001

^1^ Each treatment mean was derived from 6 birds, with 1 representative bird selected from each replicate cage; CON = basal diet control; F and WF denote diets containing the fermented and unfermented substrate mixtures, respectively; numerals 3, 5, and 7 denote dietary inclusion levels (%); L* = lightness; a* = redness; b* = yellowness. ^2^ The six dietary treatments containing the fermented or unfermented substrate mixture were analyzed as a 2 × 3 factorial arrangement with fermentation status (fermented vs. unfermented), inclusion level (3%, 5%, and 7%), and their interaction as fixed effects; CON was excluded from the factorial analysis. SEM represents the pooled standard error of a treatment-combination mean derived from the residual mean square of the factorial model. Lowercase superscripts: Within a row, means among the six dietary treatment combinations lacking a common lowercase superscript differ when the fermentation status × inclusion level interaction was significant (Tukey- or Holm-adjusted *p* < 0.05). ^†^ Indicates a difference from CON (Dunnett- or Holm-adjusted *p* < 0.05).

**Table 9:** Effects of fermentation status and dietary inclusion level of the SFVS-based substrate mixture on cooking loss, shear force, and texture profile analysis (TPA) parameters of breast muscle in yellow-feathered broilers ^1^.

Trait		CON	Fermented Substrate Mixture			Unfermented Substrate Mixture			SEM ^2^	*p*-Value		
			3F	5F	7F	3WF	5WF	7WF		Fermentation Status	Inclusion Level	Fermentation Status × Inclusion Level
	Cooking loss, %	19.03	18.49 ^b^	18.96 ^b^	18.62 ^b^	19.23 ^b^	21.08 ^a†^	21.02 ^a†^	0.32	<0.001	0.003	0.034
	Shear force, N	36.53	28.85 ^ab^	28.17 ^ab†^	33.16 ^a^	23.17 ^bc†^	30.91 ^ab^	18.45 ^c†^	1.86	<0.001	0.096	<0.001
Texture profile analysis (TPA)	Hardness, gf	336.21	273.07 ^bc^	337.08 ^abc^	367.03 ^ab^	280.53 ^bc^	191.99 ^c†^	504.76 ^a^	45.77	0.653	0.001	0.015
	Springiness	0.60	0.61	0.63	0.69 ^†^	0.59	0.58	0.67	0.02	0.133	0.001	0.831
	Cohesiveness	0.67	0.69	0.69	0.75 ^†^	0.68	0.72	0.73	0.02	0.896	0.007	0.386
	Gumminess, gf	226.34	187.15 ^bc^	234.38 ^abc^	273.76 ^ab^	191.33 ^bc^	138.32 ^c†^	370.48 ^a^	33.65	0.672	<0.001	0.028
	Chewiness, gf	135.10	114.16 ^bc^	148.26 ^abc^	189.52 ^ab†^	113.51 ^bc^	80.22 ^c†^	250.77 ^a^	22.82	0.488	<0.001	0.028
	Resilience	0.32	0.35	0.36	0.43 ^†^	0.31	0.36	0.38 ^†^	0.01	0.006	<0.001	0.059

^1^ Each treatment mean was derived from 6 birds, with 1 representative bird selected from each replicate cage; CON = basal diet control; F and WF denote diets containing the fermented and unfermented substrate mixtures, respectively; numerals 3, 5, and 7 denote dietary inclusion levels (%). ^2^ The six dietary treatments containing the fermented or unfermented substrate mixture were analyzed as a 2 × 3 factorial arrangement with fermentation status (fermented vs. unfermented), inclusion level (3%, 5%, and 7%), and their interaction as fixed effects; CON was excluded from the factorial analysis. SEM represents the pooled standard error of a treatment-combination mean derived from the residual mean square of the factorial model. Lowercase superscripts: Within a row, means among the six dietary treatment combinations lacking a common lowercase superscript differ when the fermentation status × inclusion level interaction was significant (Tukey- or Holm-adjusted *p* < 0.05). ^†^ Indicates a difference from CON (Dunnett- or Holm-adjusted *p* < 0.05).

**Table 10:** Effects of fermentation status and dietary inclusion level of the SFVS-based substrate mixture on serum antioxidant indices and hepatic enzyme activities of yellow-feathered broilers ^1^.

Sample	Variable	CON	Fermented Substrate Mixture			Unfermented Substrate Mixture			SEM ^2^	*p*-Value		
			3F	5F	7F	3WF	5WF	7WF		Fermentation Status	Inclusion Level	Fermentation Status × Inclusion Level
Serum	MDA, nmol/mL	7.00	6.93^c^	6.30 ^c†^	6.85 ^c^	7.63 ^b†^	8.14 ^ab†^	8.78 ^a†^	0.16	<0.001	0.002	0.001
	T-SOD, U/mL	212.67	221.67 ^a†^	224.00 ^a†^	212.33 ^b^	206.33 ^c†^	191.17 ^d†^	178.67 ^e†^	1.08	<0.001	<0.001	<0.001
	GSH-Px, U/mL	850.50	851.14 ^b^	899.17 ^a†^	838.31 ^b^	835.57 ^b^	827.00 ^b^	649.02 ^c†^	11.31	<0.001	<0.001	<0.001
	CAT, U/mL	4.30	13.42 ^a†^	8.25 ^b†^	8.79 ^b†^	3.64 ^c†^	3.39 ^c†^	3.34 ^c†^	0.20	<0.001	<0.001	<0.001
Liver tissue	ALT, U/g protein	27.78	24.77 ^e†^	22.57 ^f†^	26.13 ^d†^	31.80 ^c†^	35.38 ^b†^	39.52 ^a†^	0.28	<0.001	<0.001	<0.001
	AST, U/g protein	71.84	68.71	66.64 ^†^	72.67	73.08	73.37	80.40 ^†^	1.33	<0.001	<0.001	0.441
	ALP, King unit/g protein	20.14	19.37 ^d^	22.24 ^c†^	24.70 ^b†^	24.81 ^b†^	24.46 ^b†^	27.43 ^a†^	0.47	<0.001	<0.001	0.004

^1^ Each treatment mean was derived from 6 birds, with 1 representative bird selected from each replicate cage; CON = basal diet control; F and WF denote diets containing the fermented and unfermented substrate mixtures, respectively; numerals 3, 5, and 7 denote dietary inclusion levels (%); MDA = malondialdehyde; T-SOD = total superoxide dismutase; GSH-Px = glutathione peroxidase; CAT = catalase; ALT = alanine aminotransferase; AST = aspartate aminotransferase; ALP = alkaline phosphatase. ^2^ The six dietary treatments containing the fermented or unfermented substrate mixture were analyzed as a 2 × 3 factorial arrangement with fermentation status (fermented vs. unfermented), inclusion level (3%, 5%, and 7%), and their interaction as fixed effects; CON was excluded from the factorial analysis. SEM represents the pooled standard error of a treatment-combination mean derived from the residual mean square of the factorial model. Lowercase superscripts: Within a row, means among the six dietary treatment combinations lacking a common lowercase superscript differ when the fermentation status × inclusion level interaction was significant (Tukey- or Holm-adjusted *p* < 0.05). ^†^ Indicates a difference from CON (Dunnett- or Holm-adjusted *p* < 0.05).

**Table 11:** Effects of fermentation status and dietary inclusion level of the SFVS-based substrate mixture on jejunal morphometric indices of yellow-feathered broilers ^1^.

Item	CON	Fermented Substrate Mixture			Unfermented Substrate Mixture			SEM ^2^	*p*-Value		
		3F	5F	7F	3WF	5WF	7WF		Fermentation Status	Inclusion Level	Fermentation Status × Inclusion Level
Villus height (VH), μm	256.55	320.20 ^a†^	294.85 ^b†^	226.99 ^c†^	166.06 ^de†^	151.15 ^e†^	188.65 ^d†^	5.82	<0.001	<0.001	<0.001
Crypt depth (CD), μm	32.63	32.23 ^b^	24.17 ^c†^	15.50 ^d†^	24.93 ^c†^	30.25 ^b^	38.65 ^a†^	0.65	<0.001	0.053	<0.001
Villus height-to-crypt depth ratio (VH:CD)	7.86	9.93 ^b†^	12.20 ^a†^	14.64 ^a†^	6.66 ^c†^	4.50 ^d†^	4.88 ^d†^	0.50	<0.001	0.092	<0.001

^1^ Each treatment mean was derived from 6 birds, with 1 representative bird selected from each replicate cage; CON = basal diet control; F and WF denote diets containing the fermented and unfermented substrate mixtures, respectively; numerals 3, 5, and 7 denote dietary inclusion levels (%). ^2^ The six dietary treatments containing the fermented or unfermented substrate mixture were analyzed as a 2 × 3 factorial arrangement with fermentation status (fermented vs. unfermented), inclusion level (3%, 5%, and 7%), and their interaction as fixed effects; CON was excluded from the factorial analysis. SEM represents the pooled standard error of a treatment-combination mean derived from the residual mean square of the factorial model. Lowercase superscripts: Within a row, means among the six dietary treatment combinations lacking a common lowercase superscript differ when the fermentation status × inclusion level interaction was significant (Tukey- or Holm-adjusted *p* < 0.05). ^†^ Indicates a difference from CON (Dunnett- or Holm-adjusted *p* < 0.05).

## Data Availability

The raw 16S rRNA gene sequencing data generated in this study have been deposited in the National Center for Biotechnology Information (NCBI) Sequence Read Archive (SRA) under BioProject accession number PRJNA1503630. The dataset is currently under embargo and will be made publicly available upon publication. Other data supporting the findings of this study are available from the corresponding author upon reasonable request.

## Supplementary Materials

The following supporting information can be downloaded at: https://www.mdpi.com/article/10.3390/ani16213363/s1, Table S1: Factors, levels, and experimental design of the Box–Behnken response surface experiment; Table S2: Analytical methods, standards, and principal instruments used for the determination of nutrient composition and pH; Table S3: Primer sequences used for reverse transcription and quantitative PCR analysis; Table S4: Analysis of variance for the quadratic regression model of the weighted score; Table S5: Mean differences and Dunnett-adjusted simultaneous 95% confidence intervals for overall growth performance in comparisons with CON; Figure S1. Linear discriminant analysis effect size (LEfSe) cladogram of differentially abundant cecal bacterial taxa in yellow-feathered broilers fed diets containing the SFVS-based substrate mixture; Figure S2: Spearman correlation heatmap between selected differentially abundant bacterial genera and host phenotypic traits in yellow-feathered broilers.
