# Supplementary material for: Effects of Mixed-Strain Solid-State Fermentation on Mycotoxin Levels and Feeding Value of a Mixture Containing Spent Flammulina velutipes Substrate for Yellow-Feathered Broilers
Source: Animals (Basel). 2026 Oct 25;16(21):3363. doi: 10.3390/ani16213363 (PMC13761480; doi:10.3390/ani16213363)
Supplement: Supplementary file 1 [file animals-16-03363-s001.zip › animals-4633051-supplementary.pdf]

Table S1. Factors, levels, and experimental design of the Box–Behnken response surface experiment.

(A). Factors and levels

Level	A: Fermentation time (d)	B: Moisture content (%)	C: Total inoculum level (%)
-1	3	40	5
0	5	45	7
1	7	50	9

(B). Experimental design matrix and observed weighted scores

Run	A: Fermentation time (d)	B: Moisture content (%)	C: Total inoculum level (%)	Weighted score[1]
1	7	45	5	56.44
2	5	45	7	70.31
3	3	50	7	66.40
4	7	40	7	51.59
5	5	45	7	71.99
6	5	40	9	65.38
7	3	40	7	52.89
8	5	40	5	59.54
9	3	45	5	63.21
10	7	45	9	60.84
11	5	45	7	70.14
12	3	45	9	66.21
13	5	50	5	68.51
14	5	45	7	71.95
15	5	50	9	69.75
16	5	45	7	70.52
17	7	50	7	51.94

[1]Weighted scores are means of 3 independent fermentation replicates and were calculated as 0.6 × percentage reduction in ZEN concentration + 0.4 × percentage reduction in DON concentration. ZEN = zearalenone; DON = deoxynivalenol.

Table S2. Analytical methods, standards, and principal instruments used for the determination of nutrient composition and pH.

**Item**	**Analytical method**	**Standard**	**Principal instrument/model and manufacturer**
Moisture	Direct drying method	GB/T 6435-2014	DHG-9243BS-III forced-air drying oven (Shanghai CIMO Medical Instrument Manufacturing Co., Ltd., Shanghai, China)
Crude protein (CP)	Kjeldahl method	GB/T 6432-2018	KDT-08C digestion furnace (Suzhou Tianwei Instrument Co., Ltd., Suzhou, Jiangsu, China); K1100 automatic Kjeldahl analyzer (Hanon Advanced Technology Group Co., Ltd., Jinan, Shandong, China)
Crude fiber (CF)	Acid–alkali digestion method	GB/T 6434-2006	XL-2000 muffle furnace (Hebi Hengke Instrument and Meter Co., Ltd., Hebi, Henan, China)[1]
Ether extract (EE)	Soxhlet extraction method	GB/T 6433-2006	Soxhlet extraction apparatus
Gross energy (GE)	Oxygen bomb calorimetry	ISO 9831:1998	C 6000 oxygen bomb calorimeter (IKA Works Guangzhou, Guangzhou, Guangdong, China)
Amino acid composition	Amino acid analyzer method	GB/T 18246-2019	L-8900 amino acid analyzer (Hitachi High-Tech Corporation, Tokyo, Japan)
pH	Potentiometric method	DB15/T 1458-2018	PHS-3E pH meter (Shanghai INESA Scientific Instrument Co., Ltd., Shanghai, China)

[1]The XL-2000 muffle furnace was used for the ashing step in crude fiber determination.

Table S3. Primer sequences used for reverse transcription and quantitative PCR analysis.

Gene	Primer[1]	Nucleotide sequence (5′–3′)	NCBI accession number
IL1B	F	ACTGGGCATCAAGGGCTA	NM_204524.2
	R	GGTAGAAGATGAAGCGGGTC	
IL6	F	GCGAAAAGCAGAACGTCGAG	NM_204628.2
	R	TCGGGATTTATCACCATCTGCC	
LITAF	F	CCGCCCAGTTCAGATGAGTTGC	NM_204267.2
	R	GCCACCACACGACAGCCAAG	
TJP1	F	GCAGTCGTTCACGATCTCCT	XM_040706827.2
	R	TCTCTGCTTCGAAGACTGCC	
OCLN	F	TCATCGTCATCCTGCTCTGC	NM_205128.1
	R	CACGTTCTTCACCCACTCCT	
CLDN1	F	TACTCCTGGGTCTGGTTGGT	NM_001013611.2
	R	GTGCTGACAGACCTGCAATG	
BAX	F	TGGATTCTCACAGTAGGAGGATG	FJ977571.1
	R	GATGAAGCCACCCAGCAGTA	
BCL2	F	ATGACCGAGTACCTGAACCG	NM_205339.2
	R	CAAGAGTGATGCAAGCTCCC	
CASP3	F	TCCACCGAGATACCGGACTG	NM_204725.1
	R	ACAAAACTGCTTCGCTTGCT	

Table S3. Cont.

Gene	Primer[1]	Nucleotide sequence (5′–3′)	NCBI accession number
LOC768817	**F**	GAAGTTCAAGTTGCCAGTCCAC	XM_040651478.2
	**R**	GATGGGGAAGGCAACAGCCA	
AMY2A	F	TTGGAGGAGTTCAGGTTTCTC	NM_001001473.3
	R	TCCCACATGTTGAGTGGGTG	
PNLIPRP1	F	CTAGGCTGCACAATGCTTCG	NM_001277382.1
	R	TGGGCTCCAGGGTAACCTAT	
ACTB	F	CAACACAGTGCTGTCTGGTGGTA	NM_205518.2
	R	ATCGTACTCCTGCTTGCTGATCC	
GAPDH	F	ACTGTCAAGGCTGAGAACGG	NM_204305.2
	R	CATTTGATGTTGCTGGGGTC	

[1]F = forward; R = reverse.

Table S4. Analysis of variance for the quadratic regression model of the weighted score[1].

Source	Sum of squares	df	Mean square	*F*-value	*P*-value
Model	818.54	9	90.95	127.03	< 0.0001
A	97.30	1	97.30	135.90	< 0.0001
B	92.48	1	92.48	129.17	< 0.0001
C	26.21	1	26.21	36.61	0.0005
AB	43.30	1	43.30	60.47	0.0001
AC	0.4900	1	0.4900	0.6844	0.4354
BC	5.29	1	5.29	7.39	0.0298
A^2	396.05	1	396.05	553.17	< 0.0001
B^2	131.03	1	131.03	183.01	< 0.0001
C^2	0.6454	1	0.6454	0.9014	0.3740
Residual	5.01	7	0.7160	—	—
Lack of fit	1.68	3	0.5615	0.6751	0.6110
Pure error	3.33	4	0.8318	—	—
Corrected total	823.55	16	—	—	—

[1]A = fermentation time; B = moisture content; C = total inoculum level. Model fit statistics: R^2 = 0.9939, adjusted R^2 = 0.9861, and predicted R^2 = 0.9610.

Table S5. Mean differences and Dunnett-adjusted simultaneous 95% confidence intervals for overall growth performance in comparisons with CON.

Comparison[1]	Overall ADG		Overall ADFI		Overall FCR	
	MD[2] (g/d)	95% CI[2]	MD[2] (g/d)	95% CI[2]	MD[2]	95% CI[2]
3F vs. CON	2.103	(1.713, 2.492)	1.578	(0.393, 2.763)	-0.130	(-0.173, -0.086)
5F vs. CON	1.232	(0.842, 1.622)	-0.714	(-1.899, 0.470)	-0.138	(-0.181, -0.094)
7F vs. CON	0.031	(-0.359, 0.421)	-0.278	(-1.463, 0.906)	-0.014	(-0.057, 0.030)
3WF vs. CON	0.080	(-0.309,0.470)	0.025	(-1.159, 1.210)	-0.007	(-0.050, 0.037)
5WF vs. CON	-0.003	(-0.393,0.386)	-0.012	(-1.197, 1.173)	0.000	(-0.043, 0.043)
7WF vs. CON	-0.177	(-0.566,0.213)	0.954	(-0.231, 2.139)	0.054	(0.010, 0.097)

[1] Each treatment mean was derived from six replicate cages, with the replicate cage serving as the experimental unit. SFVS = spent *Flammulina velutipes* substrate; CON = basal diet control; F and WF denote diets containing the fermented and unfermented SFVS-based substrate mixtures, respectively; numerals 3, 5, and 7 denote dietary inclusion levels of the substrate mixture (%).

[2] MD = mean difference (treatment mean − CON mean); CI = confidence interval. Simultaneous 95% CIs were adjusted using Dunnett’s procedure for comparisons of each treatment with CON within each outcome.

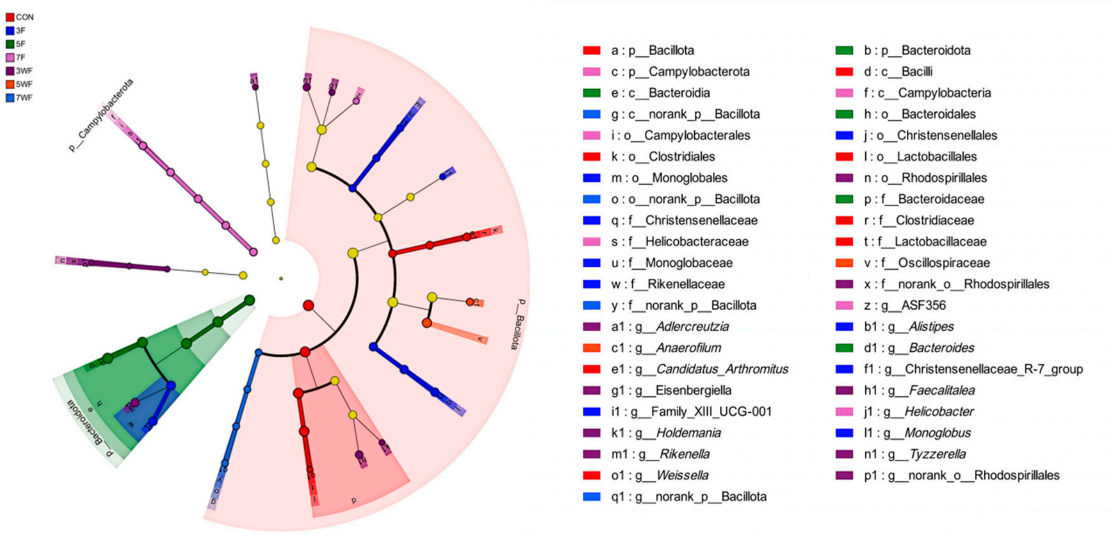

Figure S1. Linear discriminant analysis effect size (LEfSe) cladogram of differentially abundant cecal bacterial taxa in yellow-feathered broilers fed diets containing the SFVS-based substrate mixture. CON = basal diet control; F and WF denote diets containing the fermented and unfermented substrate mixtures, respectively; numerals 3, 5, and 7 denote dietary inclusion levels of the substrate mixture (%).

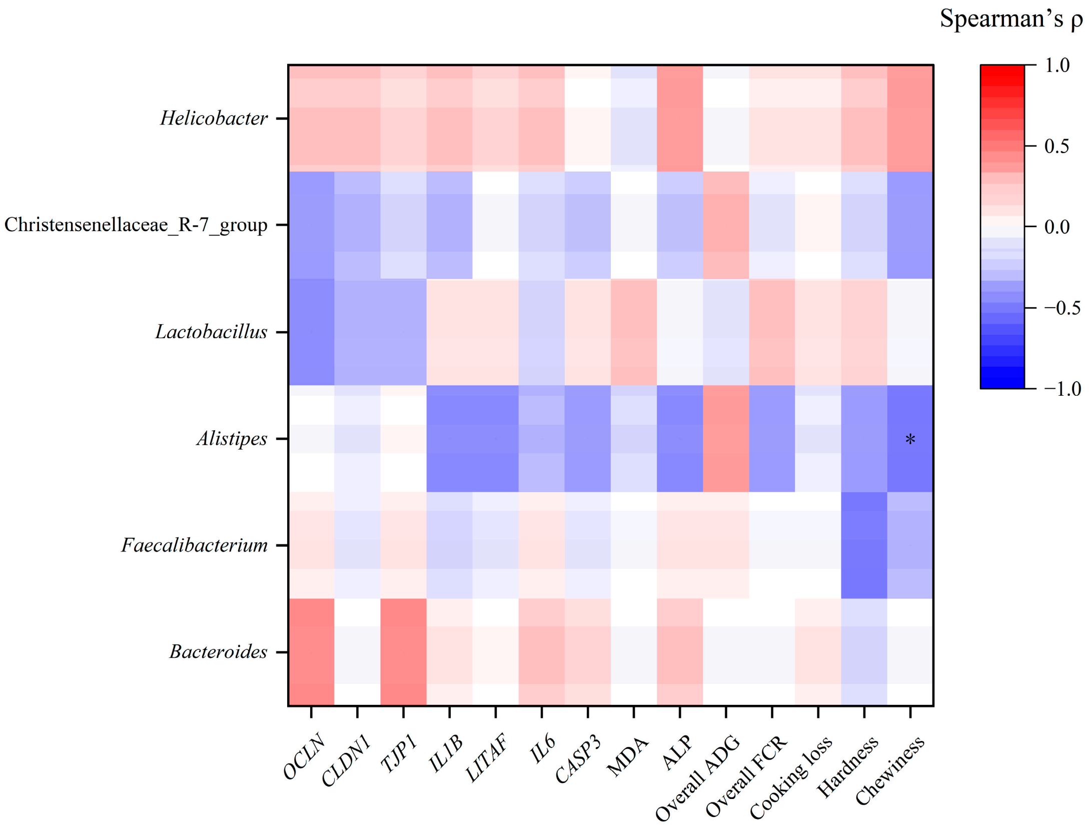

Figure S2. Spearman correlation heatmap between selected differentially abundant bacterial genera and host phenotypic traits in yellow-feathered broilers. Colors represent Spearman's rank correlation coefficients (ρ), with red and blue indicating positive and negative correlations, respectively. An asterisk (*) indicates a correlation that remained statistically significant after Benjamini–Hochberg false discovery rate (FDR) correction ($q < 0.05$). *OCLN*, occludin; *CLDN1*, claudin 1; *TJP1*, tight junction protein 1; *IL1B*, interleukin 1 beta; *LITAF*, lipopolysaccharide-induced TNF factor; *IL6*, interleukin 6; *CASP3*, caspase 3; MDA, malondialdehyde; ALP, alkaline phosphatase; ADG, average daily gain; FCR, feed conversion ratio.
